# *Clostridioides difficile* ribotypes 001 and 126 were predominant in Tehran healthcare settings from 2004 to 2018: a 14-year-long cross-sectional study

**DOI:** 10.1080/22221751.2020.1780949

**Published:** 2020-06-27

**Authors:** Masoumeh Azimirad, Marcela Krutova, Abbas Yadegar, Shabnam Shahrokh, Meysam Olfatifar, Hamid Asadzadeh Aghdaei, Warren N. Fawley, Mark H. Wilcox, Mohammad Reza Zali

**Affiliations:** a Foodborne and Waterborne Diseases Research Center, Research Institute for Gastroenterology and Liver Diseases, Shahid Beheshti University of Medical Sciences, Tehran, Iran; b Department of Medical Microbiology, Charles University, 2nd Faculty of Medicine and Motol University Hospital, Prague, Czech Republic; c European Society of Clinical Microbiology and Infectious Diseases (ESCMID) Study Group for Clostridioides Difficile (ESGCD); d Gastroenterology and Liver Diseases Research Center, Research Institute for Gastroenterology and Liver Diseases, Shahid Beheshti University of Medical Sciences, Tehran, Iran; e Basic and Molecular Epidemiology of Gastrointestinal Disorders Research Center, Research Institute for Gastroenterology and Liver Diseases, Shahid Beheshti University of Medical Sciences, Tehran, Iran; f Healthcare Associated Infections Research Group, Leeds Teaching Hospitals NHS Trust & University of Leeds, Leeds, UK

**Keywords:** *Clostridioides difficile*, ribotyping, Iran, CDI, epidemiology, clinical features, PaLoc arrangement

## Abstract

*Clostridioides difficile* infection (CDI) remains a major healthcare problem worldwide, however, little is known about CDI epidemiology in Iran. Between December 2004 and November 2018, 3649 stool samples were collected from patients in 69 hospitals and medical centres in Tehran and were cultured for the presence of *C. difficile*; isolates were characterized by PCR ribotyping and toxin genes detection. A total of 582 *C. difficile* isolates were obtained and the overall CDI prevalence was 15.9%; 290 (49.8%) cases were healthcare-associated (HA) and 292 (50.2%) cases were community-associated (CA). Of these, DNA of 513 isolates submitted for ribotyping. The ribotype and/or WEBRIBO type could be assessed in 366 (62.9%) isolates. The most frequent RTs were 001 (*n* = 75, 12.9%), 126 (*n* = 65, 11.2%) and 084 (*n* = 19, 3.3%); the toxin gene profile *tcdA*^+^*B*^+^*/cdtA*^+^*B*^+^ (*n* = 112, 19.2%) was the most common. Fifteen *C. difficile* isolates (2.6%) did not carry any toxin genes. There was no difference between frequently found RTs in HA-CDI and CA-CDI, except for RT 029 which was more likely to be associated with healthcare origin (12/15, *p*-value = 0.02). No isolate of RTs 027 or 078 was identified. Importantly, RTs 031, 038, 039, 084, 085 reported previously as RTs with an absence of toxin genes, revealed the presence of toxin genes in our study. Using Simpson’s reciprocal index of diversity, we found that RT diversity decreased as the prevalence of the RT 084 increased (*R* = −0.78, *p*-value = 0.041). Different patterns in CDI epidemiology underscore the importance of local surveillance and infection control measures in Tehran healthcare settings.

## Introduction

*Clostridioides* (*Clostridium*) *difficile* is the leading cause of nosocomial diarrhea, and is considered to be a major concern in healthcare-associated gastrointestinal infections with substantial morbidity, mortality and medical costs worldwide [1,2]. The pathogenesis of *C. difficile* infection (CDI) is mediated by the production of two large clostridial toxins, toxin A (enterotoxin) and toxin B (cytotoxin) and in some strains also by the binary toxin (CDT) [3]. The genes encoding toxin A (*tcdA*) and toxin B (*tcdB*) are part of the pathogenicity locus (PaLoc), which is a large chromosomal segment (19.6 kb) carried by toxigenic strains of *C. difficile* but lacking in non-toxigenic strains. Generally, toxigenic strains of *C. difficile* produce both toxins A and B (TcdA^+^ TcdB^+^), although some strains produce toxin B only [4].

Interestingly, recent data from non-hospital settings suggest that the incidence of community-associated *C. difficile* infection (CA-CDI) is now on the rise but underestimated [5]. In hospital-acquired *C. difficile* infection (HA-CDI), an older population of patients with comorbidities and previous antimicrobial therapy is more likely to be infected, whereas CA-CDI most-likely affects a younger population without previous antimicrobial use [5,6]. However, asymptomatic carriage of *C. difficile* is also common in healthcare settings and may provide a potential source for onward transmission of CDI, and could account for many unexplained cases [7].

The severity of CDI and its unfavourable clinical outcome is influenced by several factors including recent antimicrobial therapy, surgical and nonsurgical gastrointestinal procedures, prior hospitalization, length of hospital stay, immunocompromised status and admission to an intensive care unit (ICU) [8–10]. The rate of CDI recurrences, either as relapses or reinfections, varies from 10 to 30% with increasing rates of recurrence with each subsequent episode[11,12]. Genetic characteristics of the *C. difficile* isolates and the host’s immune response have been suggested to influence recurrence risk, CDI severity, and mortality [13,14].

*C. difficile* strains have been intensively characterized and display a largely diverse population structure in various geographic regions of the world [15]. Over the past twenty years, the emergence and spread of so-called “hypervirulent” *C. difficile* ribotype (RT) 027 (B1/NAP1) has dramatically changed the epidemiology of CDI in Europe and North America [16,17].

In order to monitor the emergence of new RTs or identify a common RT cluster in a suspected CDI outbreak, effective CDI surveillance requires the collection of epidemiological data that includes the characterization of causative *C. difficile* strains and a capillary gel-based electrophoresis (CE-ribotyping), which is the recommended typing method [18,19]. Information on the molecular epidemiology of CDI in Iran, especially with a longitudinal perspective, is limited [20–22]. Therefore, in order to obtain data on CDI epidemiology and distribution of *C. difficile* RTs in Tehran healthcare settings, and to identify the risk factors for CDI development in Iranian population, we performed a 14-year-long cross-sectional study on patients with diarrhea between December 2004 and November 2018.

## Methods

### Study design and patients

This study was undertaken at the Department of Anaerobic Bacteriology in the Research Institute for Gastroenterology and Liver Diseases (RIGLD) in Tehran, Iran. Participating patients were referred from 69 different hospitals and medical centres across 13 districts in Tehran. Fecal specimens were collected from 3649 hospitalized patients and outpatients from whom at least one sample had been submitted to the laboratory between December 2004 and November 2018 for investigation of suspected CDI based on clinical symptoms and the *C. difficile* strain carrying at least one toxin gene. A CDI origin was determined according to the European Centre for Disease Prevention and Control (ECDC) CDI surveillance criteria [19]. The CA-CDI cases were defined as those patients that developed CDI symptoms in the community or within 48 h or less after hospital admission. These patients must not have been discharged from a health-care facility in the previous 12 weeks. HA-CDI cases were defined as a patient with the onset of CDI symptoms that occurred more than 48 h after admission or less than 4 weeks after discharge from a health care facility or hospital [23]. The following clinical details were recorded for all subjects: patient demographics; antibiotic and medication history; laboratory data; and underlying health conditions.

### C. difficile culture and identification

The freshly collected stool samples were delivered to the Anaerobic Laboratory within 2 h of collection. All samples were cultured on cycloserine-cefoxitin-fructose agar (CCFA, Mast Group Ltd., Merseyside, UK) supplemented with 7% horse blood under anaerobic conditions of 85% N_2_, 10% CO_2_ and 5% H_2_ (Anoxomat® Gas Exchange System, Mart Microbiology BV, Lichtenvoorde, Netherlands) at 37°C for 48–72 h after an alcohol shock treatment. A presumptive identification of *C. difficile* colonies was based on their typical white-grey, non-hemolytic morphology on agar plates, Gram staining, and the characteristic horse manure odour. Suspected colonies were further identified by PCR on 16S rDNA gene as previously described [24,25]. The isolates were then frozen at −70°C in brain heart infusion broth (BHIB) with 20% glycerol until further analyses.

### C. difficile DNA extraction

*C. difficile* crude genomic DNA was extracted from the grown colonies on CCFA plates using QIAamp® DNA Mini Kit (Qiagen, Hilden, Germany) in accordance with the manufacturer’s instructions. The DNA concentration was determined by NanoDrop® ND-1000 spectrophotometer (Thermo Scientific, Waltham, MA, USA) and DNA integrity was assessed by electrophoresis on 0.8% (w/v) agarose gels. Extracted DNA samples were stored at −20°C until used for PCR experiments.

### Detection of toxin genes and PaLoc arrangements

The presence of *C. difficile* toxin genes and the PaLoc organization was investigated as previously described [24–26]. The oligonucleotide sequences and details of PCR conditions are shown in Supplementary Table S1. Toxigenic *C. difficile* strain RIGLD 141 (*tcdA*^+^; KF840582, *tcdB*^+^; KF840583, *cdtA*; KM047901, *cdtB*; KM047900) and *C. difficile* ATCC 700057 (*tcdA*^-^/*tcdB*^-^) were used as the reference strains. The genetic organization of various PaLoc patterns in *C. difficile* isolates was drawn using Edraw Max software version 9 (https://www.edrawsoft.com). The PaLoc genetic arrangement of *C. difficile* 630 (NC_009089.1) was used as the reference strain.

### Capillary electrophoresis ribotyping

A capillary electrophoresis (CE) PCR ribotyping was performed at the Department of Medical Microbiology, Motol University hospital, Prague, Czech Republic according to consensus PCR ribotyping protocol [18]. The CE ribotyping profiles were compared with the WEBRIBO database [27]. Unrecognized CE ribotyping profiles, where at least two *C. difficile* isolates revealed the same CE ribotyping profile, were compared with the Leeds *C. difficile* reference database (more than 800 profiles).

### Statistical analysis

Statistical analyses were performed using SPSS version 21 (IBM Corp., Armonk, NY, USA) and Microsoft Excel 2016. Chi-square and Fisher’s exact tests were used to compare categorical variables. We used logistic regression to identify factors associated with CDI. We first used the univariate analysis to select candidate variables (with a *p*-value below 0.25) to perform multivariable logistic regression analysis. An odds ratio (OR) with a 95% confidence interval (CI) was calculated for all associations analyzed. Generally, a *p*-value of less than 0.05 was considered to be statistically significant. We also used ggplot2 and plotly R software packages 3.6.0 for Windows to draw figures and graphs.

## Results

### The prevalence of CDI and clinical characteristics of CDI patients

Between December 2004 and November 2018, 3649 stool samples were investigated for the presence of *C. difficile*. A total of 582 (15.9%) *C. difficile* isolates were recovered; 315 (54.1%) samples were derived from females and 267 (45.9%) from males; the mean age of the patients was 42.47 years (median 39.5 years, aged from <2 to 96 years). For CDI origin, 290 (49.8%; mean age 43.27 years, median 41 years) cases were HA-CDI, and 292 (50.2%; mean age 41.68 years, median 38 years) cases were CA-CDI. Demographic data and clinical characteristics of 3649 suspected patients with CDI enrolled in the study are summarized in Supplementary Table S2.

In comparing CDI and non-CDI patients, no statistical significance (*p*-value >0.05) was found for age, gender, underlying disease, previous antibiotic and/or gastric acid suppressant use, previous hospitalization, frequency and consistency of stools, duration of diarrhea or the hospital ward on admission.

The majority of CDI patients (*n* = 391, 76.2%) had a history of prior antibiotic usage within the last three months. The most common antibiotics used among the CDI patients were metronidazole (192/391), ciprofloxacin (100/391), vancomycin (57/391), carbapenems (72/391), cephalosporines (53/391), amikacin (45/391), chloramphenicol (32/391), co-amoxiclav (9/391), trimethoprim/sulfamethoxazole (7/391), and clindamycin (6/391). No difference in previous antibiotic consumption was observed between HA-CDI and CA-CDI, (*p*-value >0.05). The details and frequency of different antibiotics used among the patients are presented in Supplementary Table S3.

### The prevalence rate of CDI over the study period

The prevalence rate of CDI differs significantly in Tehran since, over the study period, an increase in CDI was observed during 2014 (Supplementary Figure S1A). The lowest rates of CDI were recorded in 2004 (12/582, 2.1%) compared to the highest rate in 2017 (79/582, 13.6%). The first increase of CDI rate was seen in 2011 (62/582 isolates) followed by 2014 and 2017 (77/582 and 79/582 isolates). The CDI prevalence rate varied between different age groups, with the highest rate in patients aged >65 years (416, 71.3%) and the lowest in children aged <19 years (55, 9.4%); seven children were younger than two years of age (Supplementary Table S2 and Figure S1B). The prevalence of CDI in elderly patients aged 65 to ≥85 years was 19.1% (111/582) and the prevalence of HA-CDI and CA-CDI during the study period is shown in Supplementary Figure S2. There was an increased prevalence peak for HA-CDI in 2011 (35/290 cases), while CA-CDI increased notably in 2014 (62/292 cases).

### Univariate and multivariate analysis and the risk of CDI

Logistic regression analyses demonstrated that the following factors were associated with CDI: the patients’ age; stool consistency; endocrine disease; skin disorder; hospital wards; and outpatients (Table 1). A univariate analysis revealed that the following were significant risk factors and determinants for CDI: adult and elderly age groups; circulatory system disease; endocrine disease; blood cancer; bone marrow transplant (BMT); psychiatric wards; and out-patients. However, in a multivariate analysis (Table 1), all of the following were associated significantly with CDI: age; loose stools; endocrine disease; skin disorder; BMT ward; and outpatients.

**Table 1.** Univariate and multivariate logistic regression predictors of CDI among studied patients.

Epidemiological characteristics	Univariate analysis	*p*-value	Multivariate analysis	*p*-value
	OR (95% CI)		OR (95% CI)	
Children				
<2	1.00		1.00	
2–11	1.31 (0.55–3.07)	0.532	1.186 (0.49–2.84)	0.479
12–18	1.38 (0.54–3.51)	0.489	1.41 (0.54–3.71)	**0**.**033**
Adult				
19–64	2.40 (1.10–5.22)	**0**.**027**	2.42 (1.07–5.47)	**0**.**011**
Elderly				
65–74	2.94 (1.28–6.71)	**0**.**010**	3.06 (1.29–7.21)	**0**.**029**
75–84	2.68 (1.14–6.31)	**0**.**023**	2.68 (1.1.10–6.51)	**0**.**001**
≥85	4.37 (1.75–10.90)	**0**.**002**	4.70 (1.81–12.18)	0.701
Gender				
Female	1.00		1.00	
Male	0.876 (0.73–1.04)	0.145	0.897 (0.74–1.08)	0.255
Stool consistency				
Watery	1.00		1.00	
Loose	0.83 (0.69–1.01)	0.066	0.82 (0.67–0.99)	**0**.**04**
Mucous-filled	1.42 (0.29–6.8)	0.659	1.77 (0.33–9.3)	0.497
Formed	1.43 (0.82–2.49)	0.197	1.44 (0.81–2.5)	0.206
Medication exposure				
Antimicrobials				
Consumed	1.00	** **	1.00	** **
Not-consumed	1.03 (0.82–1.29)	0.780	0.87 (0.68–1.13)	0.317
Unknown	1.03 (0.78–1.36)	** **	1.01 (0.75–1.35)	0.925
IBD drugs	1.00	** **	1.00	** **
Immunosuppressant	1.07 (0.44–2.60)	0.874	1.09 (0.44–2.72)	0.842
Corticosteroids	0.38 (0.15–0.94)	0.038	0.44 (0.17–1.12)	0.087
Anti-TNF	0.32 (0.38–2.70)	0.297	0.36 (0.42–3.09)	0.355
Corticosteroids + immunosuppressant	0.5 (0.17–1.46)	0.207	0.48 (0.16–1.46)	0.199
Corticosteroids + anti-inflammatory	0.41 (0.08–1.98)	0.270	0.34 (0.07–1.67)	0.187
Anti-inflammatory + immunosuppressant	0.77 (0.22–2.60)	0.678	0.72 (0.21–2.48)	0.613
Corticosteroids + anti-inflammatory + immunosuppressant	0.26 (0.03–2.17)	0.215	0.25 (0.03–2.11)	0.205
Chemotherapeutic agents	1.20 (0.37–3.85)	0.749	1.96 (0.53–7.24)	0.312
Duration of diarrhea				
2–3 days	1.00		1.00	
<1 day	0.80 (0.54–1.18)	0.263	0.80 (0.53–1.21)	0.305
1 day	0.85 (0.63–1.15)	0.302	0.88 (0.64–1.20)	0.434
>3 days	0.93 (0.77–1.14)	0.539	0.94 (0.76–1.15)	0.5599
Defecation (times/day)				
3–5	1.00		1.00	
1–2	1.34 (0.68–2.62)	0.388	1.29 (0.64–2.5)	0.471
5–8	1.12 (0.91–1.37)	0.260	1.11 (0.9–1.37)	0.312
8–10	1.38 (0.81–2.35)	0.229	1.55 (0.89–2.69)	0.114
>10	1.12 (0.85–1.48)	0.403	1.03 (0.83–1.38)	0.832
Comorbidities				
Digestive system diseases	1.00		1.00	
Respiratory system disease	0.96 (0.58–1.57)	0.876	1.13 (0.65–1.94)	0.658
Circulatory system disease	0.42 (0.51–1.65)	**0**.**046**	0.54 (0.22–1.22)	0.170
Genitourinary system disease	0.92 (0.51–1.65)	0.791	1.04 (0.54–2)	0.893
Endocrine disease	1.85 (1.14–3)	**0**.**012**	2.2 (1.32–3.99)	**0**.**003**
Blood cancer	0.59 (0.44–0.79)	**0**.**001**	1.07 (0.66–1.72)	0.772
Solid cancer	0.43 (0.1–1.83)	0.255	0.59 (0.13–2.63)	0.494
Immunodeficiency disorder	1.17 (0.58–2.37)	0.643	2.14 (0.97–4.68)	0.057
Neurological disorder	1 (0.65–1.55)	0.978	1.31 (0.78–2.19)	0.297
Skin disorder	4.95 (0.69–35.26)	0.110	12.8 (1.09–151.84)	**0**.**043**
Fever with unknown cause	0.75 (0.41–1.37)	0.359	0.80 (0.43–1.49)	0.489
Allergic disorder	4.95 (0.30–79.35)	0.258	5.88 (0.34–99.85)	0.220
Surgical procedure	1.55 (0.78–3.09)	0.206	1.95 (0.91–4.20)	0.085
Accident	2.47 (0.22–27.37)	0.460	4.93 (0.40–59.73)	0.210
Others	1.23 (0.56–2.70)	0.592	2.09 (0.84–5.16)	0.109
Hospital wards				
Gastroenterology	1.00		1.00	
Infectious disease	1 (0.71–1.4)	0.981	1.37 (0.92–2.04)	0.117
Internal	0.91 (0.68–1.2)	0.521	0.99 (0.72–1.38)	0.956
Surgery	1.12 (0.72–1.74)	0.586	1.00 (0.61–1.65)	0.975
Intensive care unit (ICU)	0.81 (0.55–1.19)	0.300	0.71 (0.45–1.14)	0.161
Pediatrics	0.86 (0.35 –2.09)	0.754	1.19 (0.43–3.33)	0.729
Oncology	0.73 (0.52–1.01)	0.064	0.88 (0.53–1.45)	0.621
Coronary care unit (CCU)	1.19 (0.54–2.62)	0.660	0.87 (0.37–2.05)	0.762
Urology	2.46 (0.73–8.2)	0.145	3.04 (0.84–11.03)	0.090
Gynecology	0.98 (0.43–2.24)	0.970	0.38 (0.38–2.19)	0.848
Endocrinology	1.89 (0.66–5.36)	0.230	1.35 (0.44–4.17)	0.596
Nephrology	0.91 (0.47–1.77)	0.798	0.78 (0.36–1.67)	0.528
Bone marrow transplant (BMT)	0.30 (0.16–0.55)	**0**.**001**	0.37 (0.17–0.79)	**0**.**010**
Orthopedics	0.82 (0.23–2.80)	0.752	0.41 (0.10–1.65)	0.211
Psychiatrics	3.93 (1.04–14.78)	**0**.**042**	3.47 (0.83–14.40)	0.086
Cardiology	1.03 (0.34–3.07)	0.949	1.11 (0.35–3.52)	0.853
General medicine	1.96 (0.37–10.21)	0.420	1.93 (0.35–10.48)	0.442
Out-patients	1.51 (1.09–2.08)	**0**.**012**	1.67 (1.19–2.33)	**0**.**002**
Laboratory tests				
Leukocytosis	0.82 (0.65–1.04)	0.116	0.93 (0.72–1.21)	0.626
Neutropenia	0.64 (0.46–0.87)	0.006	0.89 (0.61–1.29)	0.551

CDI, *Clostridioides difficile* infection; IBD, inflammatory bowel disease; OR, odds ratio; CI, confidence interval. We first used the univariate analysis to select candidate variables (with *p*-value below 0.25) to perform multivariable logistic regression analysis. An OR with a 95% CI was calculated for all associations analyzed. Generally, statistical significance was declared for *p*-value less than 0.05 as shown in bold. Data were analyzed with ggplot2 and plotly R software packages.

### C. difficile toxin gene detection

Of the 582 *C. difficile* isolates tested, 566 (97.2%) carried at least one toxin gene. In 19 isolates, a partial deletion in *tcdB* (A^+^B^-^) was observed. The remaining 16 (2.7%) isolates were negative for both toxin A and B genes and also negative for binary toxin genes except one isolate (*tcdA^-^, tcdB^-^, cdtA^+^B^+^*)*.* A total of 117 (20.1%) isolates were found to carry the binary toxin genes (*cdtA*^+^ *B*^+^). These isolates were either *tcdA^+^B^+^* or *tcdA^+^B^-^*, except one isolate which was *cdtA*^+^ *B*^+^ (Table S2).

In total, five different toxin gene profiles were identified among the toxin gene(s) carrying *C. difficile* isolates: 544 (93.5%) had *tcdA*^+^ *B*^+^/*cdtA*^-^ *B*^-^; 112 (19.2%) had *tcdA*^+^ *B*^+^/*cdtA*^+^ *B*^+^; 22 (3.8%) carried *tcdA*^+^; 4 (0.7%) *tcdA*^+^/*cdtA*^+^ *B*^+^; and one isolate was positive for the binary toxin (*cdtA*^+^ *B*^+^) only.

## Capillary PCR ribotyping

**Figure 1.**
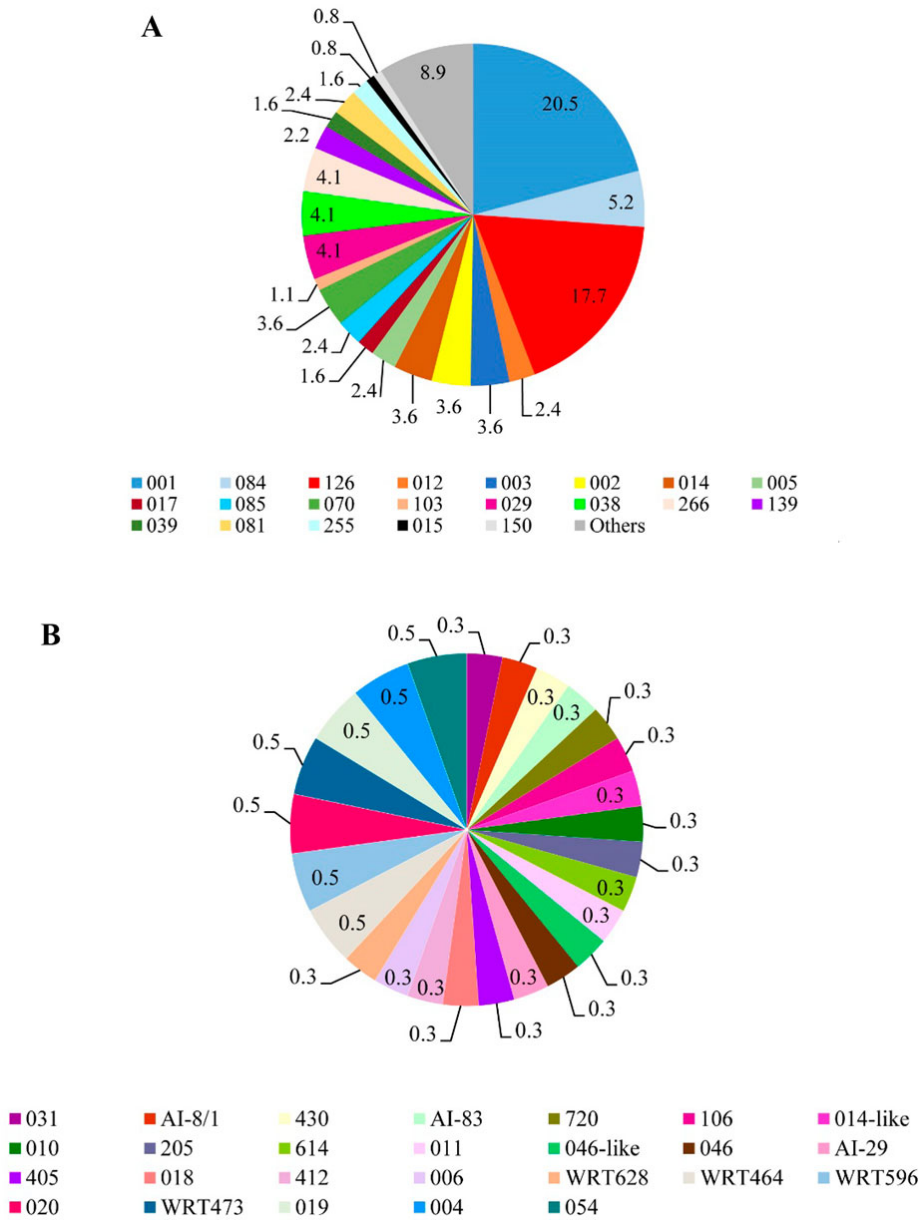
Frequency of PCR ribotypes of 366 *C. difficile* isolates during the study period in Tehran. (A) Most commonly identified *C. difficile* PCR ribotypes. (B) Other *C. difficile* PCR ribotypes; ribotypes found at a frequency of less than 10 isolates in a year were grouped into the others category.

Of the 582 *C. difficile* isolates, the DNA of 513 *C. difficile* isolates was sent for capillary electrophoresis ribotyping. The ribotype and/or WEBRIBO type could be assessed in 366 (62.9%) of *C. difficile* isolates. The most frequent RTs were 001 (*n* = 75, 20.5%; *tcdA^+^B^+^*) and 126 (*n* = 65, 17.7%; *tcdA^+^B^+^*, *cdtA*^+^ *B*^+^) followed by RTs: 084 (*n* = 19, 5.2%; *tcdA^+^B^+^*, *cdtA*^+^ *B*^+^), 029 (*n* = 15, 2.6%; *tcdA^+^B^+^*), 038 (*n* = 15, 4.1%; *tcdA^+^B^+^*), 266 (*n* = 15, 4.1%; *tcdA^+^B^+^*), 002; 003; 014; 070 (*n* = 13, 3.6% each; *tcdA^+^B^+^*). The other CE PCR ribotyping profiles did not exceed 10 isolates per profile (2.7%). No isolate of RTs 027 or 078 was recognized. One hundred and forty-seven isolates remained unrecognized. The frequency of the most common RTs and others during the study period are shown in Figure 1A and 1B. In addition, the distribution of various toxin genes profiles in *C. difficile* RTs is summarized in Supplementary Table S4 and Figure S3.

## The distribution of C. difficile RTs over the study period

**Figure 2.**
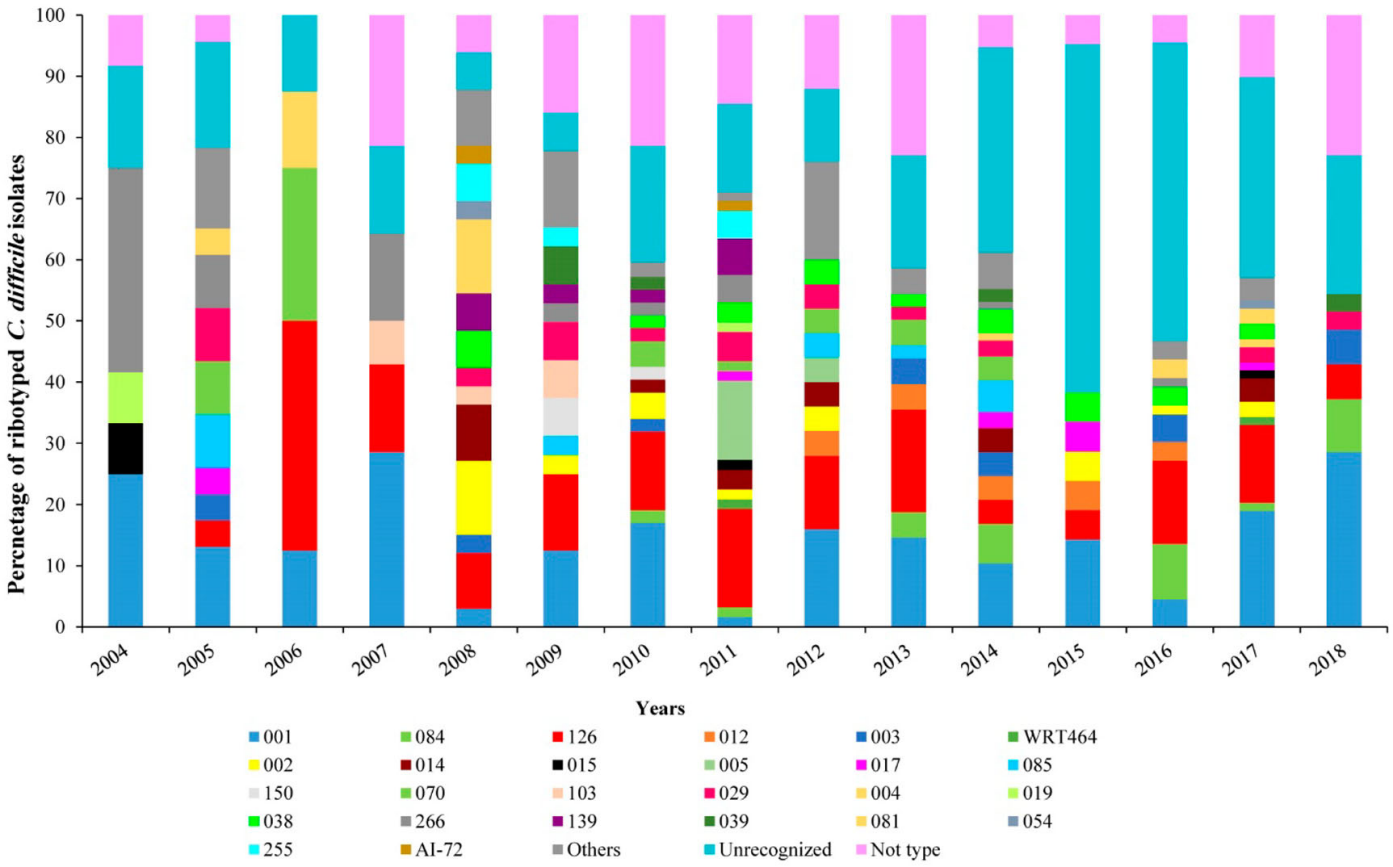
Distribution of *C. difficile* ribotypes during the study period (2004–2018). Each CE-ribotyping profile is represented by a different colour.

As shown in Figure 2, the distribution of RTs varied noticeably over the study period. These results showed a striking increase in the frequency of RTs 001, 003, 084, 126, 017, and 038, along with a concomitant decrease in RTs 012, 002, 014, 070, 103, 029, 266, and 081 over the same time period; the RTs 001 and 126 were detected in all years of the study. The incidence of RT 001 increased recently in 2017 and 2018 (15/75 and 10/75 isolates) whereas for RT 126, the first peak of increased incidence could be seen in 2011 (10/65 isolates) followed by 2016 and 2017 (9/65 and 10/65 isolates). The first incidence of RT 084 was seen in 2010 (1/19 isolates) followed by further incidences in 2014 and 2016 (5/19 and 6/19 isolates).

## The distribution of C. difficile RTs in patient age groups

**Figure 3.**
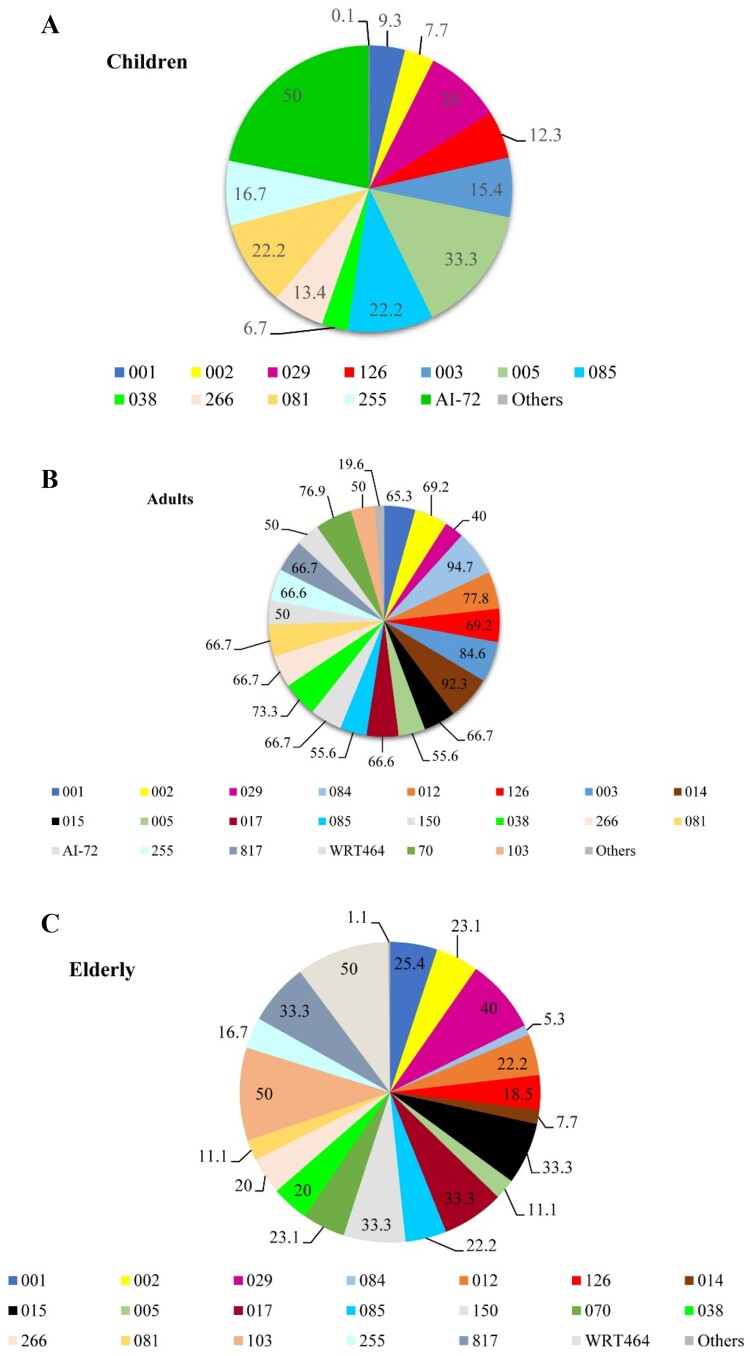
Distribution of *C. difficile* ribotypes according to patient age groups. (A) Distribution of PCR ribotypes of *C. difficile* isolates in children (<2–18 years). (B) Distribution of PCR ribotypes of *C. difficile* isolates in adults (19–64 years). (C) Distribution of PCR ribotypes of *C. difficile* isolates in elderly (65 to ≥85 years).

To establish if the diversity and distribution of *C. difficile* RTs varied according to the patient’s age, we analyzed their distribution in three patient age groups: children, <2–18 years (*n* = 36); adults, 19–64 years (*n* = 259); and the elderly, 65 to ≥85 years (*n* = 71). The distribution of RTs differed in each patient age group with a predominance of RTs 126 (*n* = 8, 12.3%) and 001 (*n* = 7, 9.3%) in children, RTs 001 (*n* = 49, 65.3%), 126 (*n* = 45, 69.2%), 084 (*n* = 18, 94.7%), 014 (*n* = 12, 92.3%), 003 (*n* = 11, 84.6%) and 038 (*n* = 11, 73.3%) in adults RTs 001 (*n* = 19, 25.4%), 126 (*n* = 12, 18.5%) and 029 (*n* = 6, 40%) in the elderly. Using Simpson’s reciprocal index analysis of diversity, an increase of RT diversity with patient age was also observed (Figure 3A–C).

## The diversity of C. difficile RTs in hospital wards

The distribution of *C. difficile* RTs in different hospital wards is shown in Supplementary Figure S4. *C. difficile* RTs were distributed in the gastroenterology, internal, surgery, ICU and oncology wards. The distribution of the RT 017 was seen only in the gastrointestinal and internal wards. The RTs 150, 004 and 020 were the most common RTs in internal, oncology units and out-patients; RT 139 was identified only in the psychiatric ward.

## The distribution of C. difficile toxin genes profiles and RTs in HA-CDIs and CA-CDIs

The distribution of the toxin genes profiles of *C. difficile* isolates in HA-CDIs and CA-CDIs is shown in Supplementary Figure S5. Five toxin genes profiles were identified in HA-CDI and CA-CDI cases: *tcdA*^+^ *B^+^* (93.8%, 272/290 vs. 93.6%, 272/290); *tcdA^+^B^-^* (2.6%, 8/290 vs. 4.8%, 14/290); *tcdA*^+^ *B^+^/cdtA*^+^ *B^+^* (18.6%, 54/290 vs. 18.8%,55/290); *tcdA*^+^ */cdtA*^+^ */B*^+^ (0.7%, 2/290 vs. 0.7%, 2/292); and *tcdA*^-^ *B^-^/cdtA*^+^ *B^+^* (0.3%, 1/290 vs. 0). By CDI origin, no difference was found between frequently found RTs and HA- and CA-CDIs, except for RT 029 which was more likely to be the cause of HA-CDI (12/15, *p*-value = 0.02). The distribution of frequently found *C. difficile* RTs in HA- and CA-CDIs is presented in Table 2.

**Table 2.** Distribution of frequently found *C. difficile* RTs in HA-CDI and CA-CDI in inpatients and outpatients enrolled in this study.

*C*. difficile PCR RT	HA-CDI, *n *= 290 (%)	CA-CDI, *n *= 292 (%)
001	35 (12.1)	40 (13.7)
126	34 (11.7)	31 (10.6)
084	7 (2.4)	12 (4.1)
029	12 (4.1)*	3 (1.0)
038	4 (1.3)	11 (3.8)
266	10 (3.5)	5 (1.7)
002	8 (2.8)	5 (1.7)
003	6 (2.1)	7 (2.4)
014	6 (2.1)	7 (2.4)
070	9 (3.1)	4 (1.4)
Others	59 (20.3)	51 (17.5)
Unrecognized	62 (21.4)	85 (29.1)
Not typed	38 (13.1)	31 (10.6)

RT, ribotype; HA-CDI, healthcare-associated *Clostridioides difficile* infection; CA-CDI, community-associated *Clostridioides difficile* infection. RT 029 was significantly associated with HA-CDI (*p-value = 0.02).

## The diversity of C. difficile RTs across districts of Tehran

**Figure 4.**
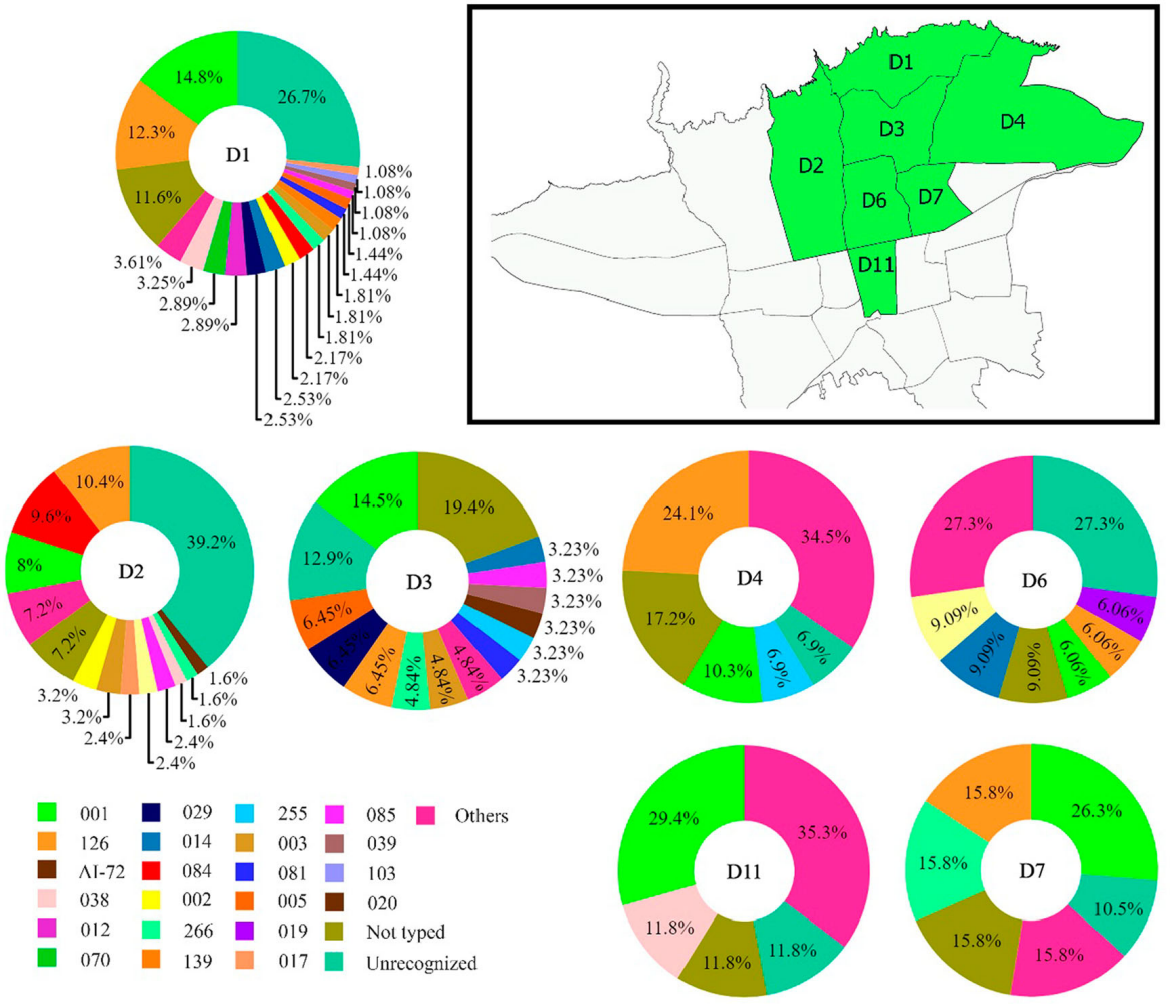
Geographical distribution of *C. difficile* PCR ribotypes across different districts of Tehran. Pie charts represent the proportion of PCR ribotypes per 7 districts (D1–D4, D6, D7 and D11) of Tehran. The text on the map and in the centre of pie charts indicates the district number of typed isolates in Tehran. The percent of total numbers for the different ribotypes in the study districts are shown on each pie chart.

The distribution of *C. difficile* RTs across different districts of Tehran is shown in Figure 4. Many of the most commonly isolated RTs were found across districts 1, 2 and 3. The RTs 001 and 126 were found almost across all districts involved in this study.

## The relationship between RT diversity and the prevalence of RTs 001, 126, and 084

**Figure 5.**
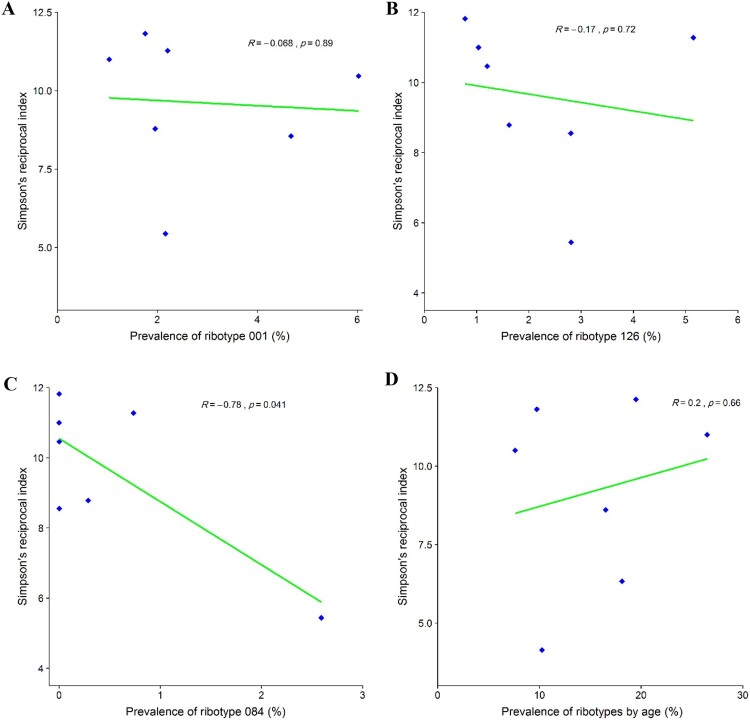
Relationship between prevalence of the most frequently found *C. difficile* PCR ribotypes in Tehran (RT 001, RT 126 and RT 084) and diversity of other ribotypes using Simpson’s reciprocal index. (A) Simpson’s reciprocal index of diversity for RT 001. (B) Simpson’s reciprocal index of diversity for RT 126. (C) Simpson’s reciprocal index of diversity for RT 084. (D) Simpson’s reciprocal index of RTs diversity with patient age.

Given that the RTs 001, 126, and 084 were identified as the most common RTs in Tehran, Simpson’s reciprocal index of diversity was used to investigate the relationship between the prevalence of these RTs with others. It was found that the RT diversity decreased as the prevalence of the RT 084 increased (*R* = 0.78, *p*-value = 0.041), Figure 5A-C. Our data suggest that districts with a high prevalence of RT 084 have a lower overall RT diversity than districts with a low prevalence of RT 084. We found that the number of unique ribotypes identified increased with patient age as shown in Figure 5D. When comparing two age groups, 41 individual RTs were isolated in patients aged 18 to <65 years, while 23 were identified in patients ≥81 years. Analysis of Simpson’s reciprocal index of diversity showed that, overall, the RT diversity was higher in patients aged ≥81 years (Simpson’s reciprocal index: 9.61) than in those aged 18 to <65 years (Simpson’s reciprocal index: 8.64).

## Paloc integrity analysis

To analyze the intactness of PaLoc, a multiplex PCR assay was implemented for 568 isolates of *C. difficile*. It should be noted that 14 isolates failed to give a PCR product for interpretation of their PaLoc integrity. Based on PCR amplifications, 16 unique groups of PaLoc arrangement were found among the studied isolates. The intact PaLoc containing *cdu2*^+^/*tcdR^+^*/*tcdA*^+^/*tcdB*^+^/*tcdE*^+^/*tcdA*^+^/*tcdC*^+^/*cdd3*^+^ genes was observed in 345/568 (59.8%) of the isolates. Genetic organization of the PaLoc in *C. difficile* isolates are illustrated in Supplementary Figure S6. The majority of RTs (40/49, 81.6%) harboured a *cdu2*^+^/*tcdR^+^*/*tcdA*^+^/*tcdB*^+^/*tcdE*^+^/*tcdA*^+^/*tcdC*^+^/*cdd3*^+^ gene arrangement. Among common RTs, of 75 isolates belonging to the RT 001, 53/75 (70.7%) carried an intact PaLoc; of the 19 isolates belonging to RT 084, 12/19 (63.2%) harboured an intact PaLoc. In addition, of the 64 isolates belonging to RT 126, 53/64 (82.8%) harboured an intact PaLoc. The frequency of PaLoc patterns among *C. difficile* isolates in relation to the different RTs is presented in Supplementary Table S5.

## Discussion

To the best of our knowledge, this is the first and largest, long-term, cross-sectional study to date on the epidemiology of CDI in Tehran healthcare settings across a large timeframe (from 2004 to 2018) that addresses the clinical features and molecular characteristics of *C. difficile*. In this study, the prevalence of CDI showed a fluctuating trend with the highest peaks in 2014 and 2017 and with an equal proportion of HA- and CA-CDIs; these data do not suggest, however, the emergence of an outbreak and/or the spread of certain hypervirulent *C. difficile* RTs. This finding is supported by the ribotyping of *C. difficile* isolates which did not reveal common RT clusters at a particular time or in a particular healthcare facility. We also found a difference in the patterns of CDI epidemiology, particularly in the prevailing RTs and their toxin genes profiles, than that reported previously in Europe and the USA [28–30].

In our study the prevalence of CDI and the distribution of the causative RTs differed greatly between hospitals in various districts of Tehran. Compared to previous data from Iran, a noticeable heterogeneity was observed among published studies particularly in terms of the study population and the prevalence of CDI that varied from 6.14% to 52% [20–22,31–34]. Compared to other countries, the prevalence of CDI in our study (15.9%) was lower than that reported in Europe, America and the Middle East [28,35–37].

Our ribotyping results showed that the molecular epidemiology of *C. difficile* was diverse and varied across Tehran healthcare settings; the RTs 001, 126 and 084 were the most frequently found. Compared to other Iranian studies, the different RTs were shown to be predominant at different time periods in hospitalized adults. The predominance of RTs 078 and 126 were found in Isfahan and Tehran single medical centres, between 10/2000 and 3/2011 and 1/2011 and 8/2011 respectively [31,38]. The latest data from 6/2016 to 11/2017 identified the predominance of RT 039 (15.8%), WEBRIBO types AI-12 (10.52%) and AI-21 (10.52%) among clinical and non-clinical isolates in three Tehran tertiary care hospitals [39]. The study from Shiraz in Iran identified only one isolate carrying genes for all three toxins out of 45 isolates investigated, while in our study this toxin gene profile was the most common (19.2%) [40].

In comparison to other Middle East countries, a diverse distribution of RTs was reported in this region. In Kuwait, geographically close to Iran, the predominant RTs were 097 and 078 which accounted for about 40% of all isolates in the intensive-therapy units (ITUs) in 2003 [41]. In a recent study conducted in Kuwait, RTs 139 (31.4%), 097 (20%) and 070 (17.1%) were reported as predominant among CA-CDI, while RTs 002 (20%), 001 (18.9%), 126 (12.6%), and 003 (10.8%) were the most frequent among the HA-CDI [42]. In Lebanon, *C. difficile* was isolated in 82.9% (107/129) of stool samples of symptomatic patients at a tertiary care university hospital, in which RT 014 (16.8%) predominated, followed by RT 002 (9.3%), RT 106 (8.4%) and RT 070 (6.5%) [43]. In a national survey of the molecular epidemiology of *C. difficile* in Israel, toxigenic *C. difficile* isolates were recovered in 208 out of 217 samples (95.8%), and RT 027 (31.8%) was the most common type [44].

However, over the past twenty years, the emergence and spread of so-called “hypervirulent” *C. difficile* RT 027 (B1/NAP1) dramatically changed the CDI epidemiology in Europe and North America [16,17]. In our study, RT 027 was not identified although a large number of isolates were characterized. In previous Iranian studies, the presence of RT 027 was identified only in the study by Khosdel et al. in children aged five years and younger [21].

Based on Simpson’s reciprocal index of diversity, we found a significant correlation between RT 084 prevalence and overall ribotype diversity, suggesting that RT 084 may be more successful at outcompeting other such ribotypes that have epidemic potential. There are very limited data on the prevalence RT 084, and this ribotype has been reported rarely in the developed countries. However, in the isolates from Ghana (40%, *n* = 6/15) and Algeria (36.4%, *n* = 4/11) in Africa, RT 084 was the most prevalent and with an equal distribution between symptomatic patients and asymptomatic controls. In addition, all were found to be nontoxigenic and resistant to erythromycin and ciprofloxacin [45–47]. When comparing the age groups, the overall ribotype diversity was higher in patients aged ≥81 years which is consistent with the results from a multicentre study performed in Europe [48].

Surprisingly, several RTs identified in our study carried toxin genes (RTs 031, 038, 039, 084, 085) but in other studies an absence of the toxin genes in these ribotypes was identified [28,45–51]. The differences in PaLoc arrangements in certain RTs were also noted previously. Kouhsari et al. observed that, among six human *C. difficile* isolates of RT 039 cultured between 6/2016 and 11/2017, only one isolate carried toxin B (*tcdB*) and five of them were also *tcdA*-positive [22]. In contrast, *C*. *difficile* isolates belonging to RT 039, derived from patients in Kuwait, did not carry toxin genes [41]. The difference in toxigenic genes profiles in *C. difficile* isolates of RT 053 recovered from river water samples was also noted by Zidaric et al. [52]. Unexpectedly, these *C. difficile* isolates did not carry toxin genes compared to the reference human RT 053 isolate that were positive for genes for toxin A and B (*tcdA* and *tcdB*). These observations are supported by data from the study of Dingle et al. [53], describing the acquisition and loss of the PaLoc DNA in whole genome data of *C. difficile* isolates from different multilocus sequence type clades.

In our study, a significant number of *C. difficile* isolates remained unrecognized. Unfortunately, only DNA samples were provided for capillary electrophoresis ribotyping and thus new RTs could not be assessed because of a lack of a corresponding *C. difficile* strain. The other limitation of our study was the absence of the recommended algorithm for CDI testing. In our study, CDI was defined by the presence of diarrhoea and *C. difficile* strain carrying at least one toxin gene, it is not certain, therefore, that each sample represents a true episode of CDI.

Several studies have described different risk factors for developing CDI including being elderly, prior antibiotic use, prior use of gastric acid suppressants, non-selective NSAID, a previous hospital stay or nursing-home admission, IBD and some other co-morbidities [10,54–58]. Among these factors, prior antibiotic exposure and old age have been documented as the major risk factors associated with complicated or recurrent diseases [8,10,14]. In our study, using multivariate analysis, we found that almost all age groups were equally at risk of developing CDI. We did not find an association between the use of certain antimicrobials and the risk of CDI, possibly because of the large number of *C. difficile* negative patients who had a prior history of antibiotic usage in our study.

It has been also reported that use of certain antibiotics, especially fluoroquinolones, has been associated with infections by RT 027 compared with those who were infected with other RTs [59,60]. Moreover, Bauer et al. found that an infection with RT 018 or RT 056 was associated with a complicated disease outcome [28]. We did not find any similar associations, because no CDI infections resulted from RT 027 and RT 056, and, in this study, there was only one RT 018 infection. In contrast, we found a significant association between infections with RT 029 and HA-CDI which has not been reported elsewhere.

## Conclusion

In summary, this study presents the first CDI surveillance data in Tehran healthcare settings, in which the molecular epidemiological characteristics, prevalence and risk factors of *C. difficile* were determined across a large timespan. Different patterns in CDI epidemiology were observed in Tehran healthcare settings. The previous consumption of antimicrobials and gastric acid suppressors were not significant risk factors for the development of CDI in Iranian patients. The greater diversity and lack of significant prevalence of a particular ribotype in HA-CDIs suggests a limited contribution of healthcare settings to the transmission of *C. difficile*. The toxin gene profiles *tcdA^+^B^+^/cdtA^+^B^+^* were the most common and RTs 001, 126 and 084 were the most frequently identified. Importantly, some RTs previously identified with an absence of PaLoC carried toxin genes. Further investigations by whole genome sequencing and cytotoxicity assay are needed for those strains.

## Supplementary Material

Supplementary_data.docx
