# Supplementary material for: Clostridioides difficile ribotypes 001 and 126 were predominant in Tehran healthcare settings from 2004 to 2018: a 14-year-long cross-sectional study
Source: Emerg Microbes Infect. 2020 Jun 27;9(1):1432–43. doi: 10.1080/22221751.2020.1780949 (PMC7473134; doi:10.1080/22221751.2020.1780949)
Supplement: Supplementary_data.docx [file TEMI_A_1780949_SM4607.docx]

**Table S1.** Oligonucleotide sequences used in this study.

| **Target gene** | **Primer** | **Sequencing** | **Product size (bp)** | **PCR conditions** | **Reference** |
| --- | --- | --- | --- | --- | --- |
| 16S rRNA | PS13  PS14 | GGAGGCAGCAGTGGGGAATA  TGACGGGCGGTGTGTACAAG | 1062 | 94°C 10 min,  35 cycles  (94°C 50 sec;  54°C 40 sec;  72°C 50 sec),  72°C 5 min | 24 |
| *tcdA* | tcdA-F3345  tcdA-R3969 | GCATGATAAGGCAACTTCAGTGGTA  AGTTCCTCCTGCTCCATCAAATG | 629 |  |  |
| *tcdB* | tcdB-F5670  tcdB-R6079A  tcdB-R6079B | CCAAARTGGAGTGTTACAAACAGGTG  GCATTTCTCCATTCTCAGCAAAGTA  GCATTTCTCCGTTTTCAGCAAAGTA | 410 |  |  |
| *cdtA* | cdtA-F739A  cdtA-F739B  cdtA-R958 | GGGAAGCACTATATTAAAGCAGAAGC  GGGAAACATTATATTAAAGCAGAAGC  CTGGGTTAGGATTATTTACTGGACCA | 221 |  |  |
| *cdtB* | ctdB-F617  cdtB-R878 | TTGACCCAAAGTTGATGTCTGATTG  CGGATCTCTTGCTTCAGTCTTTATAG | 262 |  |  |
| *cdd3* | Tim 6  Struppi 6 | TCCAATATAATAAATTAGCATTCC  GGCTATTACACGTAATCCAGATA | 622 | 95°C 5 min,  35 cycles  (95°C 1 min;  57.1°C 1 min;  72°C 1 min),  72°C 5 min | 26 |
| *tcdC* | Tim 2  Struppi 2 | GCACCTCATCACCATCTTCAA  TGAAGACCATGAGGAGGTCAT | 345 |  |  |
| *tcdD* | Tim 3  Struppi 3 | AAAGCGATGCTATTATAGTCAAA  CCTTATTAACAGCTTGTCTAGAT | 300 |  |  |
| *tcdE* | Tim 1  Struppi 1 | GTTTAAGTGCAATAAAAAGTCGTA  GGTAATCCACATAAGCACATATT | 262 |  |  |
| *cdu2* | Tim 5  Struppi 5 | CCACAGATGCTTTTAGCAGGAA  TCCAATCACTGCTCCAGCTAT | 162 |  |  |

**Table S2.** Demographic data and clinical characteristics of 3649 suspected patients with CDI and their toxin profiles enrolled in this study.

| **Epidemiological characteristics** | **CDI testing and toxin profiles** | | | | | | | | | | |
| --- | --- | --- | --- | --- | --- | --- | --- | --- | --- | --- | --- |
|  | **CDI-negative (n=3067)** | **CDI-positive (n=582)** | **HA-CDI (n=290)** | **CA-CDI (n=292)** | ***tcdA^+^B^+^***  **(n=544)** | ***tcdA^+^B^-^***  **(n=22)** | ***tcdA^-^B^-^***  **(n=16)** | ***cdtA^+^B^+^***  **(n=117)** | ***tcdA^+^B^+^/cdtA^+^B^+^***  **(n=112)** | ***tcdA^+^B^-^/cdtA^+^B^+^***  **(n=4)** | ***tcdA^-^B^-^/cdtA^+^B^+^***  **(n=1)** |
| **Mean age ±SD** | 38.61 ± 22.01 | 42.47 ± 21.66 | 43.27 ± 22.37 | 41.68 ± 20.94 | 43.49 ± 22.16 | 36.32 ± 17.76 | 42.93 ± 28.21 | 39.56 ± 19.59 | 40.30 ± 19.03 | 22.75 ± 13.93 |  |
| **Children** |  |  |  |  |  |  |  |  |  |  |  |
| <2 | 85 (2.8) | 7 (1.2) | 6 (2.1) | 1 (0.3) | 6 (1.1) | 0 | 1 (6.3) | 0 | 0 | 0 | 0 |
| 2-11 | 296 (9.6) | 32 (5.5) | 15 (5.2) | 17 (5.8) | 30 (5.5) | 1 (4.5) | 1 (6.3) | 11 (9.4) | 10 (8.9) | 1 (25) | 0 |
| 12-18 | 140 (4.6) | 16 (2.7) | 5 (1.7) | 11 (3.8) | 14 (2.6) | 1 (4.5) | 1 (6.3) | 1 (0.8) | 1 (0.9) | 0 | 0 |
| **Adult** |  |  |  |  |  |  |  |  |  |  |  |
| 19-64 | 2104 (68.6) | 416 (71.3) | 198 (68.3) | 218 (74.7) | 390 (71.7) | 18 (81.8) | 8 (50) | 90 (76.9) | 86 (76.8) | 3 (75) | 1 (100) |
| **Elderly** |  |  |  |  |  |  |  |  |  |  |  |
| 65-74 | 223 (7.3) | 54 (9.2) | 33 (11.4) | 21 (7.2) | 50 (9.2) | 1 (4.5) | 3 (18.8) | 8 (6.8) | 8 (7.1) | 0 | 0 |
| 75-84 | 158 (5.1) | 35 (6.1) | 25 (8.6) | 10 (3.4) | 33 (6.1) | 1 (4.5) | 1 (6.3) | 5 (4.3) | 5 (4.5) | 0 | 0 |
| ≥85 | 61 (2) | 22 (3.8 | 8 (2.8) | 14 (4.8) | 21 (3.7) | 0 | 1 (6.3) | 2 (1.7) | 2 (1.8) | 0 | 0 |
| **Gender** |  |  |  |  |  |  |  |  |  |  |  |
| Female | 1559 (50.9) | 315 (54.1) | 152 (52.4) | 163 (55.8) | 290 (53.3) | 14 (63.6) | 11 (68.8) | 67 (57.3) | 62 (55.4) | 4 (100) | 1 (100) |
| Male | 1508 (49.1) | 267 (45.9) | 138 (47.6) | 129 (44.2) | 254 (46.7) | 8 (36.4) | 5 (31.3) | 50 (42.7) | 50 (44.6) | 0 | 0 |
| **Stool consistency** |  |  |  |  |  |  |  |  |  |  |  |
| Watery | 1806 (58.9) | 362 (62.2) | 182 (62.8) | 180 (61.6) | 338 (62.1) | 15 (68.2) | 9 (56.3) | 80 (68.4) | 77 (68.7) | 3 (75) | 0 |
| Loose | 1195 (39) | 201 (34.5) | 102 (35.2) | 99 (33.9) | 189 (34.7) | 6 (27.3) | 6 (37.5) | 34 (29) | 32 (28.6) | 1 (25) | 1 (100) |
| Mucous-filled | 7 (0.2) | 2 (0.3) | 5 (1.7) | 12 (4.1) | 2 (0.4) | 0 | 0 | 3 (2.6) | 3 (2.7) | 0 | 0 |
| Formed | 59 (1.91) | 17 (3) | 1 (0.3) | 1 (0.3) | 15 (2.8) | 1 (4.5) | 1 (6.3) | 0 | 0 | 0 | 0 |
| Bloody | 559 (18.3) | 139 (23.5) | 64 (22.1) | 75 (25.7) | 122 (22.4) | 8 (36.4) | 9 (56.3) | 31 (26.5) | 30 (26.8) | 0 | 1 (100 |
| **WBC in stool** |  |  |  |  |  |  |  |  |  |  |  |
| 0-1 | 2792 (91) | 526 (90.4) | 261 (90) | 265 (90.8) | 489 (89.9) | 22 (100) | 15 (93.8) | 108 (92.3) | 103 (91.9) | 4 (100) | 1 (100) |
| 2-5 | 159 (5.2) | 26 (4.5) | 15 (5.2) | 11 (3.8) | 25 (4.6) | 0 | 1 (6.3) | 5 (4.3) | 5 (4.5) | 0 | 0 |
| >5 | 63 (2.1) | 20 (3.4) | 7 (2.4) | 13 (4.5) | 20 (3.7) | 0 | 0 | 3 (2.6) | 3 (2.7) | 0 | 0 |
| Many | 53 (1.7) | 10 (1.7) | 7 (2.4) | 3 (1) | 10 (1.8) | 0 | 0 | 1 (0.8) | 1 (0.9) | 0 | 0 |
| **Hospitalization (in the last month)** | 471 (17.6) | 111 (19.2) | - | - | 83 (15.3) | 2 (9.1) | 5 (31.3) | 21 (18) | 20 (17.3\8) | 1 (25) | 0 |
| **Medication exposure** |  |  |  |  |  |  |  |  |  |  |  |
| **Antimicrobials** |  |  |  |  |  |  |  |  |  |  |  |
| Consumed | 2083 (67.8) | 391 (76.2) | 197 (67.9) | 194 (66.4) | 378 (69.4) | 6 (27.3) | 7 (43.8) | 79 (67.5) | 76 (67.9) | 2 (50) | 1 (100) |
| Not-consumed | 614 (19.9) | 119 (20.4) | 56 (19.3) | 64 (21.9) | 106 (19.5) | 7 (31.8) | 7 (43.8) | 27 (23.1.) | 27 (24.1) | 0 | 0 |
| Unknown | 370 (14.3) | 72 (12.4) | 37 (12.8) | 34 (11.6) | 60 (11) | 9 (40.9) | 2 (12.5) | 11 (9.4) | 9 (8) | 2 (50) | 0 |
| **Gastric acid suppressants** |  |  |  |  |  |  |  |  |  |  |  |
| Anti-acid drug | 563 (18.3) | 97 (16.7) | 65 (22.4) | 32 (11) | 18 (3.3) | 0 |  | 4 (3.4) | 4 (3.6) | 0 | 0 |
| H_2_-blocker | 50 (1.6) | 14 (2.4) | 8 (2.8) | 6 (2.1) | 12 (2.2) | 1 (4.5) | 1 (6.3) | 2 (1.7) | 2 (1.8) | 0 | 0 |
| Anti-acid drug + H_2_-blocker | 6 (0.2) | 1 (0.2) | 0 | 1 (0.3) | 1 (0.2) | 0 | 0 | 1 (0.8) | 1 (0.9) | 0 | 0 |
| **Anti-inflammatory** | 58 (1.9) | 20 (3.4) | 10 (3.4) | 10 (3.42) | 18 (3.3) | 2 (9.1) | 0 | 6 (5.1) | 0 | 0 | 0 |
| **Immunosuppressants** | 27 (0.9) | 10 (1.7) | 5 (1.7) | 5 (1.7) | 10 (1.8) | 0 | 0 | 5 (4.3) | 5 (4.5) | 0 | 0 |
| **Corticosteroids** | 60 (1.9) | 8 (1.4) | 6 | 2 (0.7) | 8 (1.5) | 0 | 0 | 1 (0.8) | 1 (0.9) | 0 | 0 |
| **Anti-TNF** | 9 (0.3) | 1 (0.2) | 0 | 1 (0.3) | 1 (0.2) | 0 | 0 | 0 | 0 | 0 | 0 |
| **Corticosteroids + immunosuppressants** | 29 (0.9) | 5 (0.8) | 1 (0.3) | 4 (1.4) | 5 (0.9) | 0 | 0 | 1 (0.8) | 1 (0.9) | 0 | 0 |
| **Corticosteroids + anti-inflammatory** | 14 (0.4) | 2 (0.3) | 1 (0.3) | 0 | 2 (0.4) | 0 | 0 | 0 | 0 | 0 | 0 |
| **Anti-inflammatory + immunosuppressants** | 15 (0.5) | 4 (0.7) | 3 (1) | 1 (0.3) | 3 (0.6) | 1 (4.5) | 0 | 0 | 0 | 0 | 0 |
| **Corticosteroids + anti**-**inflammatory + immunosuppressants** | 11 (0.3) | 1 (0.2) | 1 (0.3) | 0 | 1 (0.2) | 0 | 0 | 0 | 0 | 0 | 0 |
| **Chemotherapeutic agents** | 12 (0.4) | 5 (0.8) | 3 (1) | 2 (0.7) | 5 (0.9) | 0 | 0 | 1 (0.8) | 1 (0.9) | 0 | 0 |
| **Duration of diarrhea** |  |  |  |  |  |  |  |  |  |  |  |
| <1 day | 206 (6.7) | 33 (5.6) | 0 | 33 (11.3) | 33 (6.1) | 0 | 0 | 10 (8.5) | 10 (8.9) | 0 |  |
| 1 day | 368 (12.1) | 63 (10.3) | 2 (0.7) | 61 (20.9) | 53 (9.7) | 5 (22.7) | 5 (31.3) | 7 (6) | 7 (6.3) | 0 | 0 |
| 2-3 days | 1445 (47.1) | 289 (49.8) | 201 (69.3) | 88 (30.1) | 273 (50.2) | 9 (40.9) | 7 (43.8) | 58 (49.6) | 55 (49.1) | 2 (50) | 0 |
| >3 days | 1048 (34.1) | 197 (34.5) | 87 (30) | 110 (37.7) | 185 (34) | 8 (36.4) | 4 (25) | 42 (35.9) | 40 (35.7) | 2 (50) | 1 (100) |
| **Defecation (times/day)** |  |  |  |  |  |  |  |  |  |  |  |
| 1-2 | 46 (1.5) | 11 (1.7) | 5 (1.7) | 6 (2.1) | 11 (2) | 0 | 0 | 1 (0.8) | 1 (0.9) | 0 |  |
| 3-5 | 1679 (54.7) | 299 (51.6) | 152 (52.4) | 147 (50.3) | 276 (50.7) | 13 (59.1) | 10 (62.5) | 54 (46.2) | 51 (45.5) | 2 (50) | 0 |
| 5-8 | 900 (29.4) | 180 (30.9) | 88 (30.3) | 92 (31.5) | 169 (31.1) | 6 (27.3) | 5 (31.3) | 47 (40.2) | 46 (41.1) | 1 (25) | 1 (100) |
| 8-10 | 73 (2.4) | 18 (3.1) | 9 (3.1) | 9 (3.1) | 17 (3.1) | 1 (4.5) | 0 | 2 (1.7) | 2 (1.8) | 0 | 0 |
| >10 | 369 (12) | 74 (12.7) | 36 (12.4) | 38 (13) | 71 (13.1) | 2 (9.1) | 1 (6.3) | 13 (11.1) | 12 (10.7) | 1 (25) | 0 |
| **Comorbidities** |  |  |  |  |  |  |  |  |  |  |  |
| Digestive system disease | 1897 (61.9) | 383 (65.7) | 159 (54.8) | 224 (76.7) | 353 (64.9) | 18 (81.8) | 12 (75) | 88 (75.2) | 85 (75.8) | 2 (50) |  |
| Respiratory system disease | 103 (3.3) | 20 (3.5) | 17 (5.9) | 3 (1) | 20 (3.7) | 0 | 0 | 2 (1.7) | 2 (1.8) | 0 |  |
| Circulatory system disease | 70 (2.3) | 6 (1) | 4 (1.4) | 2 (0.7) | 6 (1.1) | 0 | 0 | 2 (1.7) | 2 (1.8) | 0 | 1 (100) |
| Genitourinary system disease | 75 (2.4) | 14 (2.4) | 9 (3.1) | 5 (1.7) | 13 (2.4) | 0 | 1 (6.3) | 2 (1.7) | 2 (1.8) | 0 | 0 |
| Endocrine disease | 64 (2.1) | 24 (4) | 11 (3.8) | 13 (4.5) | 22 (4.4) | 1 (4.5) | 1 (6.3) | 3 (2.6) | 3 (2.7) | 0 | 0 |
| Blood cancer | 505 (16.4) | 61 (10.5) | 41 (14.1) | 20 (6.8) | 60 (11) | 1 (4.5) | 0 | 11 (9.4) | 10 (8.9) | 0 | 0 |
| Solid cancer | 23 (0.7) | 2 (0.3) | 1 (0.3) | 1 (0.3) | 2 (0.4) | 0 | 0 | 1 (0.8) | 1 (0.9) | 1 (25) | 0 |
| Immunodeficiency disorder | 42 (1.4) | 10 (1.7) | 5 (1.7) | 5 (1.7) | 10 (1.8) | 0 | 0 | 2 (1.7) | 2 (1.8) | 0 | 0 |
| Neurological disorder | 128 (4.2) | 26 (4.5) | 19 (6.6) | 7 (2.4) | 23 (4.2) | 2 (9.1) | 1 | 3 (2.6) | 2 (1.8) | 0 | 0 |
| Skin disorder | 2 (0.06) | 2 (0.4) | 2 (0.7) | 0 | 2 (0.4) | 0 | 0 | 0 | 0 | 0 | 0 |
| Eye disorder | 1 (0.03) | 0 | 0 | 0 | 0 | 0 | 0 | 0 | 0 | 1 (25) | 0 |
| Fever with unknown cause | 85 (2.8) | 13 (2.3) | 8 (2.8) | 5 (1.7) | 12 (2.2) | 0 | 1 (6.3) | 1 (0.8) | 1 (0.9) | 0 | 0 |
| Neoplasm | 2 (0.06) | 0 | 0 | 0 | 0 | 0 | 0 | 0 | 0 | 0 | 0 |
| Allergic disorder | 1 (0.03) | 1 (0.2) | 1 (0.3) | 0 | 1 (0.2) | 0 | 0 | 0 | 0 | 0 | 0 |
| Surgical procedure | 35 (1.1) | 11 (1.9) | 7 (2.4) | 4 (1.4) | 11 (2) | 0 | 0 | 1 (0.8) | 1 (0.9) | 0 | 0 |
| Accident | 2 (0.06) | 1 (0.2) | 1 (0.3) | 0 | 1 (0.2) | 0 | 0 | 0 | 0 | 0 | 0 |
| Others | 32 (1) | 8 (1.4) | 5 (1.7) | 3 (1) | 8 (1.5) | 0 | 0 | 1 (0.8) | 1 (0.9) | 0 | 0 |
| **Hospital wards** |  |  |  |  |  |  |  |  |  |  |  |
| Gastroenterology | 994 (32.4) | 202 (34.7) | 94 (32.4) | 108 37) | 183 (33.6) | 11 (50) | 8 (50) | 47 (40.2) | 46 (41.1) | 1 (25) | 1 (100) |
| Infectious disease | 245 (8) | 50 (8.6) | 28 (9.7) | 22 (7.5) | 46 (8.5) | 2 (9.1) | 2 (12.5) | 8 (6.8) | 7 (6.2) | 0 | 0 |
| Internal | 437 (14.2) | 81 (13.9) | 41 (14.1) | 40 (13.70 | 79 (14.5) | 1 (4.5) | 1 (6.3) | 20 (17.1) | 20 (17.8) | 0 | 0 |
| Surgery | 122 (4) | 28 (4.8) | 21 (7.2) | 7 (2.4) | 26 (4.8) | 1 (4.5) | 1 (6.3) | 5 (4.3) | 5 (4.5) | 0 | 0 |
| Intensive care unit (ICU) | 217 (7.1) | 36 (6.1) | 24 (8.3) | 12 (4.1) | 34 (6.3) | 1 (4.5) | 1 (6.3) | 2 (1.7) | 2 (1.8) | 0 | 0 |
| Pediatrics | 34 (1.1) | 6 (1) | 0 | 6 (2.1) | 5 (0.9) | 0 | 1 (6.3) | 1 (0.8) | 1 (0.9) | 0 | 0 |
| Oncology | 343 (11.2) | 51 (8.8) | 35 (12.1) | 16 (5.5) | 51 (9.4) | 0 | 0 | 12 (10.3) | 12 (10.7) | 0 | 0 |
| Coronary care unit (CCU) | 33 (1.1) | 8 (1.4) | 7 (2.4) | 1 (0.3) | 8 (1.5) | 0 | 0 | 2 (1.7) | 2 (1.8) | 0 | 0 |
| Urology | 8 (0.2) | 4 (0.7) | 2 (0.7) | 2 (0.7) | 4 (0.8) | 0 | 0 | 0 | 0 | 0 | 0 |
| Gynaecology | 35 (1.1) | 7 (1.2) | 4 (1.4) | 3 (1) | 7 (1.3) | 0 | 0 | 2 (1.7) | 2 (1.8) | 0 | 0 |
| Endocrinology | 13 (0.4) | 5 (0.8) | 2 (0.7) | 3 (1) | 5 (0.9) | 0 | 0 | 0 | 0 | 0 | 0 |
| General medicine | 5 (0.2) | 2 (0.3) | 2 (0.7) | 0 | 2 (0.4) | 0 | 0 | 0 | 0 | 0 | 0 |
| Nephrology | 59 (1.9) | 11 (1.8) | 6 (2.1) | 5 (1.7) | 10 (1.8) | 0 | 1 (6.3) | 4 (3.4) | 4 (3.6) | 0 | 0 |
| Bone marrow transplant (BMT) | 196 (6.4) | 12 (2) | 8 (2.8) | 4 (1.4) | 12 (2.8) | 0 | 0 | 0 | 0 | 0 | 0 |
| Neonates | 1 (0.0) | 0 | 0 | 0 | 0 | 0 | 0 | 0 | 0 | 1 (25) | 0 |
| Orthopedics | 18 (0.6) | 3 (0.5) | 0 | 3 (1) | 3 (0.6) | 0 | 0 | 0 | 0 | 0 | 0 |
| Psychiatrics | 6 (0.2) | 4 (0.7) | 0 | 4 (1.4) | 3 (0.6) | 1 (4.5) | 0 | 1 (0.8) | 0 | 0 | 0 |
| Cardiology | 19 (0.7) | 8 (1.4) | 0 | 0 | 3 (0.6) | 0 | 0 | 0 | 0 | 2 (50) | 0 |
| Radiotherapy | 13 (0.4) | 0 | 0 | 0 | 0 | 1 (4.5) | 0 | 0 | 0 | 0 | 0 |
| Ear, nose, and throat (ENT) | 3 (0) | 0 | 0 | 0 | 0 | 0 | 0 | 0 | 0 | 0 | 0 |
| Maternity | 3 (0) | 0 | 0 | 0 | 0 | 0 | 0 | 0 | 0 | 0 | 0 |
| Poisoning | 1 (0) | 0 | 0 | 0 | 0 | 0 | 0 | 0 | 0 | 0 | 0 |
| Unknown | 57 (1.8) | 62 (10.6) | 3 (1) | 0 | 6 (1.1) | 0 | 0 | 13 (11.1) | 0 | 0 | 0 |
| **Out-patients** | 202 (6.6) | 4 (0.7) | 11 (38) | 51 (17.5) | 57 (10.5) | 4 (18.2) | 1 (6.3) | 0 | 11 (9.8) | 0 | 0 |
| **Laboratory tests** |  |  |  |  |  |  |  |  |  |  |  |
| Leukocytosis | 608 (19.8) | 99 (17.1) | 223 (76.9) | 250 (85.6) | 94 (17.3) | 1 (4.5) | 4 (25) | 20 (17.1) | 20 (17.8) | 0 | 0 |
| Neutropenia | 535 (17.4) | 47 (8.1) | 261 (90) | 274 (93.8) | 47 (8.3) | 0 | 0 | 11 (9.4) | 11 (9.8) | 0 | 0 |
| HIV screening | 5 (0.2) | 1 (0.2) | 0 | 1 (0.3) | 1 (0.2) | 0 | 0 | 0 | 0 | 0 | 0 |

CDI, *Clostridioides difficile* infection; HA-CDI, healthcare-associated *Clostridioides difficile* infection; CA-CDI, community-associated *Clostridioides difficile* infection.

**Table S3.** Frequency of different antibiotic patterns used among the 3649 patients involved in this study.

| **Antibiotic agents** | **CDI-Negative (n=3067)** | **CDI-Positive (n=582)** | **HA-CDI**  **(n=290)** | **CA-CDI**  **(n=292)** | **Total**  **(n=3649)** |
| --- | --- | --- | --- | --- | --- |
| AMP | 9 (0.3) |  |  |  | 9 (0.2) |
| MEM | 66 (2.1) | 3 (0.5) | 6 (0.2) |  | 75 (2)) |
| CRO | 78 (2.54) | 13 (2.2) | 5 (1.7) | 5 (1.7) | 101 (2.8) |
| AMK | 22 (0.72) | 2 (0.3) |  | 2 (0.7) | 26 (0.7) |
| AMB | 1 (0.03) |  |  |  | 1 (0.02) |
| AZM | 5 (0.16) | 2 (0.3) |  | 2 (0.7) | 9 (0.2) |
| CPL | 25 (0.81) | 32 (5.5) | 1 (0.3) | 1 (0.3) | 59 (1.6) |
| AMC | 21 (0.68) | 4 (0.7) |  | 4 (1.3) | 29 (0.8) |
| CAZ | 24 (0.78) | 2 (0.3) | 2 (0.7) | 2 (0.7) | 30 (0.8) |
| CLI | 1(0.03) | 2 (0.3) | 2 (0.7) |  | 5 (0.1) |
| CEF | 2 (0.06) |  |  |  | 2 (0.05) |
| CPE | 2 (0.06) |  |  |  | 2 (0.05) |
| CFZ | 12 (0.39) | 1 (0.2) |  |  | 13 (0.3) |
| CFM | 37 (1.2) |  | 1 (0.3) | 6 (2) | 44 (1.2) |
| SMX | 16 (0.5) | 3 (0.5) | 2 (0.7) | 1 (0.3) | 22 (0.6) |
| CIP | 191 (6.2) | 32 (5.5) | 9 (3.1) | 13 (4.4) | 245 (6.7) |
| MET | 335 (10.9) | 64 (11) | 27 (9.3) | 43 (14.7) | 469 (12.8) |
| IMP | 95 (3.1) | 17 (2.9) | 17 (5.9) | 7 (2.4) | 136 (3.7) |
| VAN | 71 (2.3) | 11 (1.9) | 7 (2.4) | 4 (1.4) | 93 (2.5) |
| PEN | 6 (0.2) |  |  |  | 6 (0.2) |
| GEN |  | 1 (0.2) |  | 1 (0.3) | 2 (0.05) |
| RIF | 1 (0.03) |  |  |  | 1 (0.02) |
| PIP/TAZ | 2 (0.06) |  |  |  | 2 (0.05) |
| ERY | 1 (0.03) |  |  |  | 1 (0.02) |
| CFL | 1 (0.03) |  |  |  | 1 (0.02) |
| CFX | 2 (0.06) |  |  |  | 2 (0.05) |
| GFM | 1 (0.03) |  |  |  | 1 (0.02) |
| CST |  |  | 1 (0.3) |  | 1 (0.02) |
| TET | 1(0.03) |  |  |  | 1 (0.02) |
| CTX | 3 (0.09) |  |  |  | 3 (0.08) |
| CTX, MET | 4 (0.13) |  |  |  | 4 (0.1) |
| CTX, VAN | 1 (0.03) |  |  |  | 1 (0.02) |
| PEN, BIS | 1 (0.03) |  |  |  | 1 (0.02) |
| SMX, CFM | 1 (0.03) |  |  |  | 1 (0.02) |
| SMX, PEN | 2 (0.06) | 1 (0.2) |  | 1 (0.3) | 4 (0.1) |
| CIP, AMK | 1 (0.03) | 1 (0.2) | 1 (0.3) |  | 3 (0.08) |
| MET, CLI | 4 (0.13) |  |  |  | 4 (0.1) |
| CIP, CPL | 3 (0.09) |  |  |  | 3 (0.08) |
| CIP, CFM | 2 (0.06) |  |  |  | 2 (0.05) |
| MEM, GM | 1 (0.03) |  |  |  | 1 (0.02) |
| MEM, IMP | 6 (0.19) |  |  |  | 6 (0.2) |
| MEM, VAN | 46 (1.5) | 5 (0.8) | 4 (1.4) | 3 (1) | 58 (1.6) |
| MEM, GEN | 1(0.03) |  |  |  | 1 (0.02) |
| MEM, CIP | 13 (0.4) | 2 (0.3) | 3 (1) |  | 18 (0.5) |
| VAN, PIP/TAZ | 5 (0.16) |  | 1 (0.3) |  | 6 (0.16) |
| VAN, MET |  |  | 1 (0.3) |  | 1 (0.02) |
| CIP, IPM | 7(0.23) | 1 (0.2) | 1 (0.3) |  | 9 (0.2) |
| MET, VAN | 138 (4.5) | 37 (6.3) | 10 (3.4) | 10 (3.4) | 195 (5.3) |
| CIP, GEN | 1 (0.03) | 1 (0.2) |  | 1 (0.3) | 3 (0.08) |
| CFM, IPM | 1 (0.03) |  |  |  | 1 (0.02) |
| AMC, CFM | 1 (0.03) |  |  |  | 1 (0.02) |
| AMC, MEM | 1 (0.03) |  |  |  | 1 (0.02) |
| AMC, SMX | 1 (0.03) |  |  |  | 1 (0.02) |
| CIP, CFM | 2 (0.06) |  |  |  | 2 (0.05) |
| CFM, IPM | 1(0.03) |  |  |  | 1 (0.02) |
| CIP, CAZ | 2(0.06) |  |  |  | 2 (0.05) |
| CRO, VAN | 1 (0.03) |  |  |  | 1 (0.02) |
| AMP, GEN | 1 (0.03) |  |  |  | 1 (0.02) |
| AMP, CLI | 1 (0.03) |  |  |  | 1 (0.02) |
| CRO, ERY |  | 1 (0.2) |  | 1 (0.3) | 2 (0.05) |
| CRO, PIP/TAZ | 1 (0.03) |  |  |  | 1 (0.02) |
| CRO, VAN | 7 (0.23) | 1 (0.2) |  | 1 (0.3) | 9 (0.2) |
| CRO, AMK | 3 (0.09) | 1 (0.2) |  | 1 (0.3) | 5 (0.1) |
| CRO, AZM | 2 (0.06) | 1 (0.2) | 1 (0.3) |  | 4 (0.1) |
| CRO, CPL | 2 (0.06) |  |  |  | 2 (0.05) |
| CRO CLI | 4 (0.13) |  |  |  | 4 (0.1) |
| CRO, CFM | 3 (0.09) |  |  |  | 3 (0.08) |
| CRO, AMC | 1 (0.03) |  |  |  | 1 (0.02) |
| CRO, CAZ |  | 1 (0.2) | 1 (0.3) |  | 2 (0.05) |
| CRO, CFZ | 1 (0.03) |  | 1 (0.3) |  | 1 (0.02) |
| CRO, IMP | 1 (0.03) |  |  |  | 1 (0.02) |
| CRO, CIP | 3 (0.09) |  |  | 1 (0.3) | 4 (0.1) |
| CRO, MET | 20 (0.65) | 12 (2.1) | 10 (3.4) | 6 (2) | 48 (1.3) |
| CRO, GEN | 1 (0.03) |  |  |  | 1 (0.02) |
| CRO, SMX | 1 (0.03) |  |  |  | 1 (0.02) |
| AMK, CAZ | 1 (0.03) |  |  | 1 (0.3) | 2 (0.05) |
| AMK, CFM | 1 (0.03) | 1 (0.2) | 1 (0.3) | 1 (0.3) | 4 (0.1) |
| AMK, CIP | 2 (0.06) | 1 (0.2) |  | 1 (0.3) | 4 (0.1) |
| AMK, IMP | 2 (0.06) |  |  |  | 2 (0.05) |
| AMK, MET | 8 (0.26) | 1 (0.2) | 1 (0.3) |  | 10 (0.3) |
| AMK, VAN | 3 (0.09) |  |  |  | 3 (0.08) |
| AMK, PIP/TAZ | 1 (0.03) |  |  |  | 1 (0.02) |
| AZM, CLI | 1 (0.03) |  |  |  | 1 (0.02) |
| CIP, CPL | 3 (0.09) |  |  |  | 3 (0.08) |
| AZM, CFM |  | 1 (0.2) | 1 (0.3) |  | 2 (0.05) |
| AZM, CIP | 1 (0.03) |  |  |  | 1 (0.02) |
| AZM, CTX |  | 1 (0.2) |  | 1 (0.3) | 2 (0.05) |
| AZM, PEN | 2 (0.06) |  |  |  | 2 (0.05) |
| CIP, AMK | 1 (0.03) | 1 (0.2) | 1 (0.3) |  | 3 (0.08) |
| CFM, IPM | 1 (0.03) |  |  |  | 1 (0.02) |
| CIP, CAZ | 2 (0.06) |  |  |  | 2 (0.05) |
| IMP, CLP |  | 1 (0.2) | 1 (0.3) |  | 2 (0.05) |
| IMP, CFM | 1 (0.03) |  | 1 (0.3) |  | 2 (0.05) |
| CPL, VAN | 1 (0.03) |  |  |  | 1 (0.02) |
| CFZ, MET |  | 1 (0.2) |  | 1 (0.3) | 2 (0.05) |
| IMP, SMX | 1 (0.03) |  |  |  | 1 (0.02) |
| CAZ, AMC | 1 (0.03) |  |  |  | 1 (0.02) |
| CAZ, IMP | 1 (0.03) |  |  |  | 1 (0.02) |
| CFX, PEN | 1 (0.03) |  |  |  | 1 (0.02) |
| CLI, AMP | 1 (0.03) |  |  |  | 1 (0.02) |
| CLI, AMC | 1 (0.03) |  |  |  | 1 (0.02) |
| CLI, CPL | 1 (0.03) |  |  |  | 1 (0.02) |
| CLI, CAZ | 1 (0.03) |  |  |  | 1 (0.02) |
| CFM, CPL | 1 (0.03) |  |  |  | 1 (0.02) |
| CLI, GM |  | 1 (0.2) |  | 1 (0.3) | 2 (0.05) |
| CLI, IMP | 2 (0.06) |  |  |  | 2 (0.05) |
| CFM, AMK | 1 (0.03) |  |  |  | 1 (0.02) |
| CIP, CPE | 2 (0.06) |  |  |  | 2 (0.05) |
| CIP, GEN | 1 (0.03) | 1 (0.2) |  | 1 (0.3) | 3 (0.08) |
| CIP, PIP/TAZ | 1 (0.03) |  |  |  | 1 (0.02) |
| CIP, VAN | 20 (0.65) | 3 (0.5) | 1 (0.3) | 2 (0.7) | 26 (0.7) |
| IMP, GEN | 1 (0.03) |  |  |  | 1 (0.02) |
| IMP, AMK | 2 (0.06) | 1 (0.2) | 1 (0.3) |  | 4 (0.1) |
| IMP, CLI | 1 (0.03) |  |  |  | 1 (0.02) |
| IMP, BIS | 1 (0.03) |  |  |  | 1 (0.02) |
| IMP, CIP | 2 (0.06) |  | 1 (0.3) |  | 3 (0.08) |
| IMP, VAN | 72 (2.3) | 9 (1.5) | 8 (2.7) | 7 (2.4) | 96 (2.6) |
| MET, CAZ | 28 (0.9) | 1 (0.2) | 1 (0.3) |  | 30 (0.8) |
| MET, AMP | 1 (0.03) |  |  |  | 1 (0.02) |
| MET, AMC | 4 (0.1) |  |  |  | 4 (0.1) |
| MET, CFM | 3 (0.09) | 1 (0.2) | 1 (0.3) |  | 5 (0.1) |
| MET, CIP | 66 (2.1) | 6 (1) | 6 (2.1) | 12 (4.1) | 90 (2.5) |
| MET, BIS | 19 (0.6) | 5 (0.8) |  | 5 (1.7) | 29 (0.8) |
| MET, PAR | 2 (0.06) |  |  |  | 2 (0.05) |
| MET, CLP | 3 (0.09) |  |  |  | 3 (0.08) |
| MET, PIP/TAZ | 5 (0.16) |  |  |  | 5 (0.1) |
| MET, CEF | 2 (0.06) |  |  |  | 2 (0.05) |
| MET, CFX | 1 (0.03) |  |  |  | 1 (0.02) |
| MET, CFP | 1 (0.03) |  |  |  | 1 (0.02) |
| MET, IMP | 39 (1.3) | 5 (3.9) | 9 (3.1) | 2 (0.7) | 55 (1.5) |
| MET, PEN | 5 (0.16) |  |  |  | 5 (0.1) |
| MET, NIT | 1 (0.03) |  |  |  | 1 (0.02) |
| MET, MEM | 35 (1.1) | 4 (0.7) | 5 (1.7) | 1 (0.3) | 45 (1.2) |
| MET, SMX | 3 (0.09) | 1 (0.2) |  |  | 4 (0.1) |
| MEM, CRO | 1 (0.03) |  |  |  | 1 (0.02) |
| MEM, CTX | 1 (0.03) |  |  |  | 1 (0.02) |
| MEM, AMB | 1 (0.03) |  |  |  | 1 (0.02) |
| MEM, AMK | 4 (0.13) |  |  |  | 4 (0.1) |
| MEM, CAZ | 1 (0.03) |  |  |  | 1 (0.02) |
| VAN, PEN | 2 (0.06) | 1 (0.2) | 1 (0.3) |  | 4 (0.1) |
| VAN, SMX | 4 (0.13) |  |  |  | 4 (0.1) |
| VAN, AMP | 3 (0.09) | 3 (0.5) | 1 (0.3) | 3 (1) | 10 (0.3) |
| VAN, AMK | 6 (0.19) | 1 (0.2) | 1 (0.3) |  | 8 (0.2) |
| VAN, CPL | 1 (0.03) |  |  |  | 1 (0.02) |
| VAN, CAZ | 2 (0.06) | 1 (0.2) | 1 (0.3) |  | 4 (0.1) |
| VAN, CLI | 1 (0.03) |  |  |  | 1 (0.02) |
| VAN, CFM | 1 (0.03) | 1 (0.2) |  | 1 (0.3) | 3 (0.08) |
| VAN, GEN |  | 1 (0.2) | 1 (0.3) |  | 2 (0.05) |
| VAN, CFL | 1 (0.03) |  |  |  | 1 (0.02) |
| VAN, IMP | 30 (0.9) | 3 (0.5) | 3 (1) | 2 (0.7) | 38 (1) |
| VAN, CIP | 6 (0.19) | 2 (0.3) | 1 (0.3) | 1 (0.3) | 10 (0.3) |
| VAN, AMB | 2 (0.06) |  |  |  | 2 (0.05) |
| IMP, CIP, VAN | 3 (0.09) | 2 (0.3) |  | 2 (0.7) | 7 (0.2) |
| IMP, AMC, CFX | 1 (0.03) |  |  |  | 1 (0.02) |
| CRO, VAN, MET | 2 (0.06) |  |  | 5 (1.7) | 7 (0.2) |
| CRO, CIP, IMP | 1 (0.03) |  |  |  | 1 (0.02) |
| CRO, MET, CIP | 7 (0.23) | 3 (0.5) |  | 5 (1.7) | 15 (0.4) |
| CRO, CLI, MET | 1 (0.03) |  |  |  | 1 (0.02) |
| CRO, CFM, CLI | 1 (0.03) |  |  |  | 1 (0.02) |
| CRO, AMC, AMK | 1 (0.03) |  |  |  | 1 (0.02) |
| CRO, AZM, IMP | 1 (0.03) |  |  |  | 1 (0.02) |
| CRO, AMC, AMP | 1 (0.03) |  |  |  | 1 (0.02) |
| CRO, CLI, GEN | 1 (0.03) |  |  |  | 1 (0.02) |
| CRO, GM, AMC | 3 (0.09) | 1 (0.2) | 1 (0.3) |  | 5 (0.1) |
| CRO, MET, AMC | 1 (0.03) | 1 (0.2) |  | 1 (0.3) | 3 (0.08) |
| CRO, CAZ, MET |  | 1 (0.2) | 1 (0.3) |  | 2 (0.05) |
| CRO, CFM, MET |  | 1 (0.2) |  | 1 (0.3) | 2 (0.05) |
| CRO, MET, SMX | 1 (0.03) |  |  | 1 (0.3) | 2 (0.05) |
| CRO, MET, VAN | 2 (0.06) | 1 (0.2) |  | 1 (0.3) | 4 (0.1) |
| CRO, MEM, CIP | 1 (0.03) |  |  |  | 1 (0.02) |
| CRO, VAN, CFM | 1 (0.03) |  |  |  | 1 (0.02) |
| CRO, VAN, MEM | 1 (0.03) |  |  |  | 1 (0.02) |
| AMK, IMP, CIP | 1 (0.03) | 0 |  |  | 1 (0.02) |
| AMK, IMP, BIS | 0 | 1 (0.2) | 1 (0.3) |  | 2 (0.05) |
| AMK, MEM, IPM | 0 | 1 (0.2) | 1 (0.3) |  | 2 (0.05) |
| AMK, VAN, IMP | 4 (0.13) |  |  | 1 (0.3) | 5 (0.1) |
| AMK, VAN, MEM | 1 (0.03) | 2 (0.3) | 2 (0.7) |  | 5 (0.1) |
| AZM, AMC, CIP | 1 (0.03) |  |  |  | 1 (0.02) |
| CFZ, IMP, VAN |  | 1 (0.2) |  | 1 (0.3) | 2 (0.05) |
| CFZ, CLI, CAZ | 1 (0.03) |  |  |  | 1 (0.02) |
| CFX, MET, NAL | 1 (0.03) |  |  |  | 1 (0.02) |
| CIP, MEM, AMK | 1 (0.03) |  | 1 (0.3) |  | 2 (0.05) |
| CIP, MEN, VAN | 3 (0.09) |  |  |  | 3 (0.08) |
| CIP, SMX, AMK |  | 1 (0.2) |  | 1 (0.3) | 2 (0.05) |
| MET, CAZ, SMX | 1 (0.03) |  |  |  | 1 (0.02) |
| MET, CAZ, VAN | 6 (0.19) | 1 (0.2) |  | 1 (0.3) | 8 (0.2) |
| CFM, AMC, AMK | 1 (0.03) |  |  |  | 1 (0.02) |
| CFM, AMC, SMX | 1 (0.03) |  |  |  | 1 (0.02) |
| CIP, GM, CFM |  | 1 (0.2) |  | 1 (0.3) | 2 (0.05) |
| CFM, AZM, MET | 2 (0.06) |  |  |  | 2 (0.05) |
| CIP, CLI, AMK |  | 1 (0.2) | 1 (0.3) |  | 2 (0.05) |
| CFM, CIP, MET | 1 (0.03) |  |  |  | 1 (0.02) |
| CAZ, AMK, CFM |  | 1 (0.2) | 1 (0.3) |  | 2 (0.05) |
| CAZ, AZM, CIP | 1 (0.03) |  |  |  | 1 (0.02) |
| CIP, AMK, AZM | 1 (0.03) |  |  |  | 1 (0.02) |
| CIP, AMK, CLI | 1 (0.03) |  | 1 (0.3) |  | 2 (0.05) |
| CIP, AMK, CLI | 1 (0.03) |  | 1 (0.3) |  | 2 (0.05) |
| IMP, MET, CLI | 1 (0.03) |  |  |  | 1 (0.02) |
| IMP, MET, PIP/TAZ | 1 (0.03) |  |  |  | 1 (0.02) |
| IMP, MEM, CIP | 2 (0.06) |  |  |  | 2 (0.05) |
| IMP, VAN, AMK | 1 (0.03) |  |  |  | 1 (0.02) |
| IMP, VAN, MEM | 3 (0.09) |  |  |  | 3 (0.08) |
| IMP, AMK, MEM | 1 (0.03) |  |  |  | 1 (0.02) |
| IMP, MET, VAN | 1 (0.03) |  | 1 (0.3) |  | 2 (0.05) |
| CIP, AMK, AZM | 1 (0.03) |  |  |  | 1 (0.02) |
| MEM, IMP, CIP | 1 (0.03) |  |  |  | 1 (0.02) |
| CIP, CLI, AMK |  | 1 (0.2) | 1 (0.3) |  | 2 (0.05) |
| SMX, TET, CIP | 1 (0.03) |  |  |  | 1 (0.02) |
| CIP, GM, CFM |  | 1 (0.2) |  | 1 (0.3) | 2 (0.05) |
| MET, MEM, CIP | 1 (0.03) | 1 (0.2) | 1 (0.3) |  | 3 (0.08) |
| MET, VAN, CLI | 1 (0.03) |  |  |  | 1 (0.02) |
| MEM, VAN, CIP | 1 (0.03) | 1 (0.2) | 1 (0.3) |  | 3 (0.08) |
| MEM, VAN, GM | 1 (0.03) | 2 (0.3) | 2 (0.7) |  | 5 (0.1) |
| VAN, CPE, AMK | 1 (0.03) |  |  |  | 1 (0.02) |
| CIP, MEM, AMK | 1 (0.03) |  | 1 (0.3) |  | 2 (0.05) |
| CIP, VAN, AMB | 1 (0.03) |  |  |  | 1 (0.02) |
| IMP, VAN, CIP |  | 1 (0.2) |  |  | 1 (0.02) |
| IMP, VAN, MET | 1 (0.03) |  |  |  | 1 (0.02) |
| MET, ERY, CIP |  | 1 (0.2) | 1 (0.3) |  | 2 (0.05) |
| MET, CST, IMP | 1 (0.03) |  |  |  | 1 (0.02) |
| MET, AMK, CAZ | 3 (0.09) | 1 (0.2) |  |  | 4 (0.1) |
| CIP, VAN, PIP/TAZ | 2 (0.06) |  |  |  | 2 (0.05) |
| CIP, GEN, CFX |  | 1 (0.2) |  | 1 (0.3) | 2 (0.05) |
| MET, AMK, CIP | 2 (0.06) | 1 (0.2) | 0 | 2 (0.7) | 5 (0.1) |
| MET, CIP, CFM |  | 1 (0.2) | 1 (0.3) | 1 (0.3) | 3 (0.08) |
| MET, CIP, IMP | 4 (0.13) | 1 (0.2) | 0 | 1 (0.3) | 6 (0.2) |
| MET, CIP, PIP/TAZ | 1 (0.03) |  |  |  | 1 (0.02) |
| MET, CIP, BIS | 2 (0.06) | 2 (0.3) |  | 1 (0.3) | 5 (0.1) |
| MET, CIP, CAZ | 1 (0.03) |  |  |  | 1 (0.02) |
| MET, CIP, MEM | 2 (0.06) |  | 1 (0.3) |  | 3 (0.08) |
| MET, IMP, AMK | 2 (0.06) |  |  |  | 2 (0.05) |
| MET, IMP, CIP | 2 (0.06) |  |  |  | 2 (0.05) |
| MET, MEM, SMX | 1 (0.03) |  |  |  | 1 (0.02) |
| MET, CIP, VAN | 1 (0.03) |  |  |  | 1 (0.02) |
| MET, CIP, AMK | 118 (3.8) | 23 (3.9) | 1 (0.3) |  | 142 (3.9) |
| MET, CAZ, CIP | 1 (0.03) |  |  |  | 1 (0.02) |
| MET, AZM, CFM | 1 (0.03) |  |  |  | 1 (0.02) |
| MET, PEN, VAN | 8 (0.26) |  |  |  | 8 (0.2) |
| MET, MEM, VAN | 6 (0.19) |  |  |  | 6 (0.2) |
| MET, SMX, AMC | 1 (0.03) | 1 (0.2) |  | 2 (0.7) | 4 (0.1) |
| MET, MEM, CLI | 1 (0.03) |  |  |  | 1 (0.02) |
| MET, PIP/TAZ, CIP | 3 (0.09) |  |  |  | 3 (0.08) |
| MEM, CIP, VAN | 3 (0.09) |  | 1 (0.3) |  | 4 (0.1) |
| MET, VAN, PIP/TAZ | 2 (0.06) | 1 (0.2) | 1 (0.3) |  | 4 (0.1) |
| MET, VAN, AMP | 1 (0.03) |  |  |  | 1 (0.02) |
| MET, VAN, AMC | 1 (0.03) |  |  |  | 1 (0.02) |
| MET, VAN, AMK | 2 (0.06) |  |  |  | 2 (0.05) |
| MET, VAN, BIS | 1 (0.03) |  |  |  | 1 (0.02) |
| MET, VAN, CFM | 1 (0.03) |  |  |  | 1 (0.02) |
| MET, VAN, CIP | 13 (0.4) | 1 (0.2) |  | 1 (0.3) | 15 (0.4) |
| MET, VAN, CTX | 1 (0.03) |  |  |  | 1 (0.02) |
| MET, VAN, GM | 5 (0.16) |  |  |  | 5 (0.1) |
| MET, VAN, IMP | 18 (0.5) | 5 (0.8) | 4 (1.4) | 1 (0.3) | 28 (7.7) |
| MET, VAN, MEM | 2 (0.06) | 1 (0.2) | 3 (1) |  | 6 (0.2) |
| MET, VAN, CAZ | 1 (0.03) |  |  |  | 1 (0.02) |
| VAN, PIP/TAZ, AMK | 1 (0.03) |  |  |  | 1 (0.02) |
| VAN, AMK, CFM | 1 (0.03) |  |  |  | 1 (0.02) |
| VAN, MEM, AMK | 2 (0.06) |  |  |  | 2 (0.05) |
| VAN, CAZ, MET |  |  |  | 1 (0.3) | 1 (0.02) |
| MEN, CIP, VAN, AMC |  | 1 (0.2) |  |  | 1 (0.02) |
| VAN, AMK, CAZ, CIP | 1 (0.03) | 1 (0.2) | 1 (0.3) |  | 3 (0.08) |
| VAN, AMB, CIP, AZM | 2 (0.06) |  |  |  | 2 (0.05) |
| MET, VAN, MEN, CAZ |  | 1 (0.2) |  | 1 (0.3) | 2 (0.05) |
| MET, VAN, CAZ, AMK | 1 (0.03) |  |  |  | 1 (0.02) |
| MET, CIP, AMK, FCZ | 1 (0.03) |  |  |  | 1 (0.02) |
| CAZ, CIP, MET, VAN | 1 (0.03) |  |  |  | 1 (0.02) |
| CRO, VAN, MET, AMK | 1 (0.03) |  |  |  | 1 (0.02) |
| CRO, SMX, MET, CIP | 1 (0.03) |  |  |  | 1 (0.02) |
| CRO, MET, BIS, IMP |  | 1 (0.2) | 1 (0.3) |  | 2 (0.05) |
| CRO, AMK, VAN, CFX | 1 (0.03) |  |  |  | 1 (0.02) |
| CRO, CFM, MET, VAN | 1 (0.03) |  |  |  | 1 (0.02) |
| CRO, MET, CIP, PEN | 1 (0.03) |  |  |  | 1 (0.02) |
| CRO, MET, CIP, VAN | 1 (0.03) |  |  |  | 1 (0.02) |
| CRO, NA, MET, GEN | 1 (0.03) |  |  |  | 1 (0.02) |
| MET, AMK, V, MEM | 1 (0.03) |  |  |  | 1 (0.02) |
| AZM, CFM, CAZ, AMP |  | 1 (0.2) |  | 1 (0.3) | 2 (0.05) |
| CLI, CIP, MET, TOB |  | 1 (0.2) | 1 (0.3) |  | 2 (0.05) |
| CIP, VAN, MET, MEM |  | 1 (0.2) | 1 (0.3) |  | 2 (0.05) |
| MET, CIP, VAN, SMX | 1 (0.03) |  |  |  | 1 (0.02) |
| MET, CAZ, VAN, AMK | 2 (0.06) |  |  |  | 2 (0.05) |
| MET, CIP, VAN, MEM | 1 (0.03) |  |  |  | 1 (0.02) |
| CRO, AMC, MET, CFM, CFX | 1 (0.03) | 1 (0.2) | 1 (0.3) | 1 (0.3) | 4 (0.1) |
| MET, CIP, AMC, CFM, CAZ | 1 (0.03) |  |  |  | 1 (0.02) |
| MET, MEM, CPE, VAN, CIP | 1 (0.03) |  |  |  | 1 (0.02) |
| MET, MEM, VAN, AMC, CAZ | 1 (0.03) |  |  |  | 1 (0.02) |
| VAN, MET, MEM, AMK, CRO |  | 1 (0.2) | 1 (0.3) |  | 2 (0.05) |
| CFZ, PEN, CAZ, VAN, IMP, AMK | 1 (0.03) |  |  |  | 2 (0.05) |
| Unknown | 378 (12.3) | 75 (12.9) | 38 (13.1) | 37 (12.7) | 528 (14.5) |
| Unused | 611 (3.1) | 119 (20.4) | 55 (18.7) | 64 (21.9) | 849 (23.3 |

HA-CDI, healthcare-associated *Clostridioides difficile* infection; CA-CDI, community-associated *Clostridioides difficile* infection, AMP, ampicillin; MEM, meropenem; CRO, ceftriaxone; AMK, amikacin; AMB, amphotericin B; AZM, azithromycin; CPL, chloramphenicol; AMC, co-amoxiclav; CAZ, ceftazidime; CLI, clindamycin; CPE, cefepime; CFZ, cefazolin; CFM, cefixime; AZM, azithromycin; SMX, trimethoprim/sulfamethoxazole; CIP, ciprofloxacin, MET, metronidazole; IMP, imipenem; VAN, vancomycin; PEN, penicillin; GEN, gentamicin; RIF, rifampicin; PIP/TAZ, piperacillin-tazobactam; ERY, erythromycin; CFL, cephalexin; GFM, gemifloxacin; CST, colistin; TET, tetracyclines; CTX, cefotaxime; BIS, bismuth; VAN, vancomycin; PAR, paromomycin; NIT, nitrofurantoin; CFX, cephalothin; TOB, tobramycin.

**Table S4.** Frequency of different ribotypes in relation to toxin profiles of 366 *C. difficile* isolates in this study.

| **Ribotypes** | **Toxin profiles** | | | | | | | **Total**  **(n=366)** |
| --- | --- | --- | --- | --- | --- | --- | --- | --- |
|  | **tcdA^+^B^+^**  **(n=544)** | **tcdA^+^B^-^**  **(n=22)** | **tcdA^-^B^-^**  **(n=16)** | **cdtA^+^B^+^**  **(n=117)** | **tcdA^+^B^+^/cdtA^+^B^+^**  **(n=112)** | **tcdA^+^B^-^/cdtA^+^B^+^**  **(n=4)** | **tcdA^-^B^-^/cdtA^+^B^+^**  **(n=1)** |  |
| 001 | 73 (13.4) | 1 (4.5) | 1 (6.3) | 6 (5.1) | 5 (4.5) | 1 (25) | 0 | 75 (20.5) |
| 002 | 13 (2.4) | 0 | 0 | 2 (1.7) | 2 (1.8) | 0 | 0 | 13 (3.6) |
| 003 | 3 (0.6) | 0 | 0 | 0 | 0 | 0 | 0 | 13 (3.6) |
| 004 | 2 (0.4) | 0 | 0 | 0 | 0 | 0 | 0 | 2 (0.5) |
| 005 | 9 (1.7) | 0 | 0 | 3 (2.6) | 3 (2.7) | 0 | 0 | 9 (2.5) |
| 006 | 1 (0.2) | 0 | 0 | 0 | 0 | 0 | 0 | 1 (0.3) |
| 010 | 1 (0.2) | 0 | 0 | 0 | 0 | 0 | 0 | 1 (0.3) |
| 011 | 1 (0.2) | 0 | 0 | 0 | 0 | 0 | 0 | 1 (0.3) |
| 012 | 9 (1.7) | 0 | 0 | 1 (0.9) | 1 (0.9) | 0 | 0 | 9 (2.5) |
| 014 | 13 (2.4) | 0 | 0 | 0 | 0 | 0 | 0 | 13 (3.6) |
| 015 | 3 (0.6) | 0 | 0 | 0 | 0 | 0 | 0 | 3 (0.8) |
| 017 | 6 (1.1) | 0 | 0 | 0 | 0 | 0 | 0 | 6 (1.6) |
| 018 | 1 (0.2) | 0 | 0 | 0 | 0 | 0 | 0 | 1 (0.3) |
| 019 | 2 (0.4) | 0 | 0 | 1 (0.9) | 1 (0.9) | 0 | 0 | 2 (0.5) |
| 020 | 2 (0.4) | 0 | 0 | 0 | 0 | 0 | 0 | 2 (0.5) |
| 029 | 15 (2.8) | 0 | 0 | 1 (0.9) | 1 (0.9) | 0 | 0 | 15 (4.1) |
| 031 | 1 (0.2) | 0 | 0 | 1 (0.9) | 1 (0.9) | 0 | 0 | 1 (0.3) |
| 038 | 15 (2.8) | 0 | 0 | 9 (7.7) | 8 (7.1) | 1 (25) | 0 | 15 (4.1) |
| 039 | 6 (1.1) | 0 | 0 | 2 (1.7) | 2 (1.8) | 0 | 0 | 6 (1.6) |
| 046 | 1 (0.2) | 0 | 0 | 0 | 0 | 0 | 0 | 1 (0.3) |
| 054 | 2 (0.4) | 0 | 0 | 0 | 0 | 0 | 0 | 2 (0.5) |
| 070 | 13 (2.4) | 0 | 0 | 2 (1.7) | 2 (1.8) | 0 | 0 | 13 (3.6) |
| 081 | 9 (1.7) | 0 | 0 | 0 | 0 | 0 | 0 | 9 (2.5) |
| 084 | 18 (3.3) | 1 (4.5) | 0 | 11 (9.4) | 11 (9.8) | 0 | 0 | 19 (5.2) |
| 085 | 9 (1.7) | 0 | 0 | 5 (4.3) | 5 (4.5) | 0 | 0 | 9 (2.5) |
| 103 | 4 (0.7) | 0 | 0 | 0 | 0 | 0 | 0 | 4 (1.1) |
| 106 | 1 (0.2) | 0 | 0 | 0 | 0 | 0 | 0 | 1 (0.3) |
| 126 | 65 (11.9) | 0 | 0 | 41 (35) | 41 (36.6) | 0 | 0 | 65 (17.8) |
| 139 | 7 (1.3) | 1 (5.3) | 0 | 1 (0.9) | 0 | 1 (25) | 0 | 8 (2.2) |
| 150 | 3 (0.6) | 0 | 0 | 0 | 0 | 0 | 0 | 3 (0.8) |
| 205 | 1 (0.2) | 0 | 0 | 0 | 0 | 0 | 0 | 1 (0.3) |
| 255 | 5 (0.9) | 0 | 1 (6.7) | 2 (1.7) | 2 (1.8) | 0 | 0 | 6 (1.6) |
| 266 | 15 (2.8) | 0 | 0 | 1 (0.9) | 1 (0.9) | 0 | 0 | 15 (4.1) |
| 405 | 1 (0.2) | 0 | 0 | 1 (0.9) | 1 (0.9) | 0 | 0 | 1 (0.3) |
| 412 | 1 (0.2) | 0 | 0 | 0 | 0 | 0 | 0 | 1 (0.3) |
| 430 | 1 (0.2) | 0 | 0 | 0 | 0 | 0 | 0 | 1 (0.3) |
| 614 | 1 (0.2) | 0 | 0 | 0 | 0 | 0 | 0 | 1 (0.3) |
| 720 | 1 (0.2) | 0 | 0 | 0 | 0 | 0 | 0 | 1 (0.3) |
| 817 | 3 (0.6) | 0 | 0 | 1 (0.9) | 1 (0.9) | 0 | 0 | 3 (0.8) |
| WRT464 | 2 (0.4) | 0 | 0 | 0 | 0 | 0 | 0 | 2 (0.5) |
| WRT473 | 1 (0.2) | 0 | 1 (6.7) | 1 (0.9) | 0 | 0 | 1 (100) | 2 (0.5) |
| WRT596 | 2 (0.4) | 0 | 0 | 0 | 0 | 0 | 0 | 2 (0.5) |
| WRT628 | 1 (0.2) | 0 | 0 | 0 | 0 | 0 | 0 | 1 (0.3) |
| AI-8/1 | 1 (0.2) | 0 | 0 | 0 | 0 | 0 | 0 | 1 (0.3) |
| AI-29 | 1 (0.2) | 0 | 0 | 0 | 0 | 0 | 0 | 1 (0.3) |
| AI-72 | 2 (0.4) | 0 | 0 | 0 | 0 | 0 | 0 | 2 (0.5) |
| AI-83 | 1 (0.2) | 0 | 0 | 1 (0.9) | 1 (0.9) | 0 | 0 | 1 (0.3) |
| 014-LIKE | 1 (0.2) | 0 | 0 | 0 | 0 | 0 | 0 | 1 (0.3) |
| 046- LIKE | 1 (0.2) | 0 | 0 | 0 | 0 | 0 | 0 | 1 (0.3) |
| Not typed | 60 (11) | 3 (13.6) | 6 (37.5) | 5 (4.3) | 5 (4.5) | 0 | 0 | 69 (18.8) |
| Unrecognized | 107 (19.7) | 15 (68.2) | 7 (43.8) | 19 (16.2) | 17 (15.2) | 1 (25) | 0 | 147 (40.2) |

**Table S5.** Frequency of PaLoc patterns of 568 *C. difficile* isolates in relation to different ribotypes.

| **RT** | **Pattern 1**  **n=345**  **(%)** | **Pattern 2**  **n=61**  **(%)** | **Pattern 3**  **n=48**  **(%)** | **Pattern 4**  **n=1**  **(%)** | **Pattern 5**  **n=25**  **(%)** | **Pattern 6**  **n=26**  **(%)** | **Pattern 7**  **n=10**  **(%)** | **Pattern 8**  **n=6**  **(%)** | **Pattern 9**  **n=32**  **(%)** | **Pattern 10**  **n=1**  **(%)** | **Pattern 11**  **n=3**  **(%)** | **Pattern 12**  **n=3**  **(%)** | **Pattern 13**  **n=2**  **(%)** | **Pattern 14**  **n=2**  **(%)** | **Pattern 15**  **n=1**  **(%)** | **Pattern 16**  **n=2**  **(%)** |
| --- | --- | --- | --- | --- | --- | --- | --- | --- | --- | --- | --- | --- | --- | --- | --- | --- |
| 001 | 53 (15.4) | 10 (18) | 4 (8.3) | 0 | 4 (16) | 1 (3.8) | 1 (10) | 0 | 1 (3.1) | 0 | 0 | 0 | 0 | 0 | 0 | 0 |
| 002 | 11 (3.2) | 2 (3.3) | 0 | 0 | 0 | 0 | 0 | 0 | 0 | 0 | 0 | 0 | 0 | 0 | 0 | 0 |
| 003 | 6 (1.8) | 3 (4.9) | 2 (4.2) | 0 | 0 | 1 (3.8) | 0 | 0 | 1 (3.1) | 0 | 0 | 0 | 0 | 0 | 0 | 0 |
| 004 | 2 (0.6) | 0 | 0 | 0 | 0 | 0 | 0 | 0 | 0 | 0 | 0 | 0 | 0 | 0 | 0 | 0 |
| 005 | 5 (1.4) | 2 (3.3) | 0 | 0 | 1 (4) | 0 | 0 | 0 | 1 (3.1) | 0 | 0 | 0 | 0 | 0 | 0 | 0 |
| 006 | 1 (0.3) | 0 | 0 | 0 | 0 | 0 | 0 | 0 | 0 | 0 | 0 | 0 | 0 | 0 | 0 | 0 |
| 010 | 1 (0.3) | 0 | 0 | 0 | 0 | 0 | 0 | 0 | 0 | 0 | 0 | 0 | 0 | 0 | 0 | 0 |
| 011 | 1 (0.3) | 0 | 0 | 0 | 0 | 0 | 0 | 0 | 0 | 0 | 0 | 0 | 0 | 0 | 0 | 0 |
| 012 | 8 (2.3) | 0 | 0 | 0 | 0 | 0 | 0 | 0 | 1 (3.1) | 0 | 0 | 0 | 0 | 0 | 0 | 0 |
| 014 | 10 (2.9) | 2 (3.3) | 0 | 0 | 1 (4) | 0 | 0 | 0 | 0 | 0 | 0 | 0 | 0 | 0 | 0 | 0 |
| 015 | 1 (0.3) | 0 | 0 | 0 | 0 | 1 (3.8) | 0 | 0 | 1 (3.1) | 0 | 0 | 0 | 0 | 0 | 0 | 0 |
| 017 | 2 (0.6) | 1 (1.6) | 0 | 0 | 1 (4) | 0 | 0 | 0 | 2 (6.3) | 0 | 0 | 0 | 0 | 0 | 0 | 0 |
| 018 | 1 (0.3) | 0 | 0 | 0 | 0 | 0 | 0 | 0 | 0 | 0 | 0 | 0 | 0 | 0 | 0 | 0 |
| 019 | 0 | 0 | 1 (2.1) | 0 | 1 (4) | 0 | 0 | 0 | 0 | 0 | 0 | 0 | 0 | 0 | 0 | 0 |
| 020 | 2 (0.6) | 0 | 0 | 0 | 0 | 0 | 0 | 0 | 0 | 0 | 0 | 0 | 0 | 0 | 0 | 0 |
| 029 | 5 (1.4) | 6 (9.8) | 2 (4.2) | 0 | 0 | 2 (7.7) | 0 | 0 | 0 | 0 | 0 | 0 | 0 | 0 | 0 | 0 |
| 031 | 0 | 0 | 0 | 0 | 0 | 0 | 0 | 0 | 1 (3.1) | 0 | 0 | 0 | 0 | 0 | 0 | 0 |
| 038 | 10 (2.9) | 1 (1.6) | 0 | 0 | 1 (4) | 0 | 0 | 0 | 3 (9.4) | 0 | 0 | 0 | 0 | 0 | 0 | 0 |
| 039 | 5 (1.4) | 0 | 0 | 0 | 0 | 1 (3.8) | 0 | 0 | 0 | 0 | 0 | 0 | 0 | 0 | 0 | 0 |
| 046 | 0 | 0 | 0 | 0 | 1 (4) | 0 | 0 | 0 | 0 | 0 | 0 | 0 | 0 | 0 | 0 | 0 |
| 054 | 2 (0.6) | 0 | 0 | 0 | 0 | 0 | 0 | 0 | 0 | 0 | 0 | 0 | 0 | 0 | 0 | 0 |
| 070 | 9 (2.6) | 2 (3.3) | 1 (2.1) | 0 | 0 | 1 (3.8) | 0 | 0 | 0 | 0 | 0 | 0 | 0 | 0 | 0 | 0 |
| 081 | 7 (2) | 2 (3.3) | 0 | 0 | 0 | 0 | 0 | 0 | 0 | 0 | 0 | 0 | 0 | 0 | 0 | 0 |
| 084 | 12 (3.5) | 4 (6.6) | 0 | 0 | 0 | 2 (7.7) | 0 | 0 | 0 | 0 | 0 | 1 (33.3) | 0 | 0 | 0 | 0 |
| 085 | 7 (2) | 2 (3.3) | 0 | 0 | 0 | 0 | 0 | 0 | 0 | 0 | 0 | 0 | 0 | 0 | 0 | 0 |
| 103 | 4 (3.5) | 0 | 0 | 0 | 0 | 0 | 0 | 0 | 0 | 0 | 0 | 0 | 0 | 0 | 0 | 0 |
| 106 | 1 (0.3) | 0 | 0 | 0 | 0 | 0 | 0 | 0 | 0 | 0 | 0 | 0 | 0 | 0 | 0 | 0 |
| 126 | 49 (14.2) | 2 (3.3) | 6 (12.5) | 0 | 1 (4) | 2 (7.7) | 0 | 0 | 4 (12.5) | 0 | 0 | 0 | 0 | 0 | 0 | 0 |
| 139 | 8 (2.3) | 0 | 0 | 0 | 0 | 0 | 0 | 0 | 0 | 0 | 0 | 0 | 0 | 0 | 0 | 0 |
| 150 | 1 (0.3) | 1 (1.6) | 0 | 0 | 0 | 1 (3.8) | 0 | 0 | 0 | 0 | 0 | 0 | 0 | 0 | 0 | 0 |
| 205 | 1 (0.3) | 0 | 0 | 0 | 0 | 0 | 0 | 0 | 0 | 0 | 0 | 0 | 0 | 0 | 0 | 0 |
| 255 | 2 (0.6) | 1 (1.6) | 1 (2.1) | 0 | 0 | 1 (3.8) | 1 (10) | 0 | 0 | 0 | 0 | 0 | 0 | 0 | 0 | 0 |
| 266 | 14 (4.1) | 1 (1.6) | 0 | 0 | 0 | 0 | 0 | 0 | 0 | 0 | 0 | 0 | 0 | 0 | 0 | 0 |
| 405 | 0 | 0 | 0 | 0 | 0 | 1 (3.8) | 0 | 0 | 0 | 0 | 0 | 0 | 0 | 0 | 0 | 0 |
| 412 | 1 (0.3) | 0 | 0 | 0 | 0 | 0 | 0 | 0 | 0 | 0 | 0 | 0 | 0 | 0 | 0 | 0 |
| 430 | 1 (0.3) | 0 | 0 | 0 | 0 | 0 | 0 | 0 | 0 | 0 | 0 | 0 | 0 | 0 | 0 | 0 |
| 614 | 1 (0.3) | 0 | 0 | 0 | 0 | 0 | 0 | 0 | 0 | 0 | 0 | 0 | 0 | 0 | 0 | 0 |
| 720 | 1 (0.3) | 0 | 0 | 0 | 0 | 0 | 0 | 0 | 0 | 0 | 0 | 0 | 0 | 0 | 0 | 0 |
| 817 | 2 (0.6) | 0 | 1 (2.1) | 0 | 0 | 0 | 0 | 0 | 0 | 0 | 0 | 0 | 0 | 0 | 0 | 0 |
| WRT464 | 1 (0.3) | 0 | 0 | 0 | 0 | 1 (3.8) | 0 | 0 | 0 | 0 | 0 | 0 | 0 | 0 | 0 | 0 |
| WRT473 | 2 (0.6) | 0 | 0 | 0 | 0 | 0 | 0 | 0 | 0 | 0 | 0 | 0 | 0 | 0 | 0 | 0 |
| WRT596 | 1 (0.3) | 0 | 1 (2.1) | 0 | 0 | 0 | 0 | 0 | 0 | 0 | 0 | 0 | 0 | 0 | 0 | 0 |
| WRT628 | 0 | 1 (1.6) | 0 | 0 | 0 | 0 | 0 | 0 | 0 | 0 | 0 | 0 | 0 | 0 | 0 | 0 |
| AI-8/1 | 1 (0.3) | 0 | 0 | 0 | 0 | 0 | 0 | 0 | 0 | 0 | 0 | 0 | 0 | 0 | 0 | 0 |
| AI-29 | 0 | 1 (1.6) | 0 | 0 | 0 | 0 | 0 | 0 | 0 | 0 | 0 | 0 | 0 | 0 | 0 | 0 |
| AI-72 | 0 | 0 | 0 | 0 | 1 (4) | 0 | 0 | 0 | 1 (3.1) | 0 | 0 | 0 | 0 | 0 | 0 | 0 |
| AI-83 | 0 | 0 | 0 | 0 | 0 | 1 (3.8) | 0 | 0 | 0 | 0 | 0 | 0 | 0 | 0 | 0 | 0 |
| 014-LIKE | 1 (0.3) | 0 | 0 | 0 | 0 | 0 | 0 | 0 | 0 | 0 | 0 | 0 | 0 | 0 | 0 | 0 |
| 046- LIKE | 0 | 0 | 0 | 0 | 0 | 0 | 0 | 0 | 1 (3.1) | 0 | 0 | 0 | 0 | 0 | 0 | 0 |
| Not typed | 33 (9.6) | 5 (8.2) | 5 (10.4) | 0 | 2 (8) | 5 (19.2) | 3 (30) | 3 (50) | 5 (15.7) | 1 (100) | 0 | 0 | 0 | 0 | 1 (100) | 0 |
| Unrecognized | 59 (17.1) | 12 (19.7) | 24 (50) | 1 (100) | 11 (44) | 5 (19.2) | 5 (50) | 3 (50) | 10 (31.3) | 0 | 3 (100) | 2 (66.7) | 2 (100) | 2 (100) | 0 | 2 (100) |

RT, ribotype; PaLoc, pathogenicity locus; Pattern 1, *tcdA/tcdB/tcdC/tcdE/tcdR/cdu/cdd3*; Pattern 2, *tcdA/tcdB/tcdC/tcdE/cdu/cdd3*; Pattern 3, *tcdA/tcdB/tcdE/tcdR/cdu/cdd3*; Pattern 4, *tcdA/tcdC/tcdR/cdu/cdd3*; Pattern 5, *tcdA/tcdB/tcdE/cdu/cdd3*; Pattern 6, *tcdA/tcdB/tcdC/tcdR/cdu/cdd3*; Pattern 7, *tcdC/tcdE/tcdR/cdu/cdd3*; Pattern 8, *tcdA/tcdC/tcdE/tcdR/cdu/cdd3;* Pattern 9, *tcdA/tcdB/tcdC/cdu/cdd3*; Pattern 10, *tcdC/tcdR/cdu/cdd3*; Pattern 11, *tcdA/tcdC/cdu/cdd3*; Pattern 12, *tcdA/tcdE/cdu/cdd3*; Pattern 13, *tcdA/tcdE/tcdR/cdu/cdd3*; Pattern 14, *tcdA/tcdC/tcdE/cdu/cdd3*; Pattern 15, *tcdE/tcdC/cdu/cdd3*; Pattern 16, *tcdC/tcdR/cdu/cdd3*.


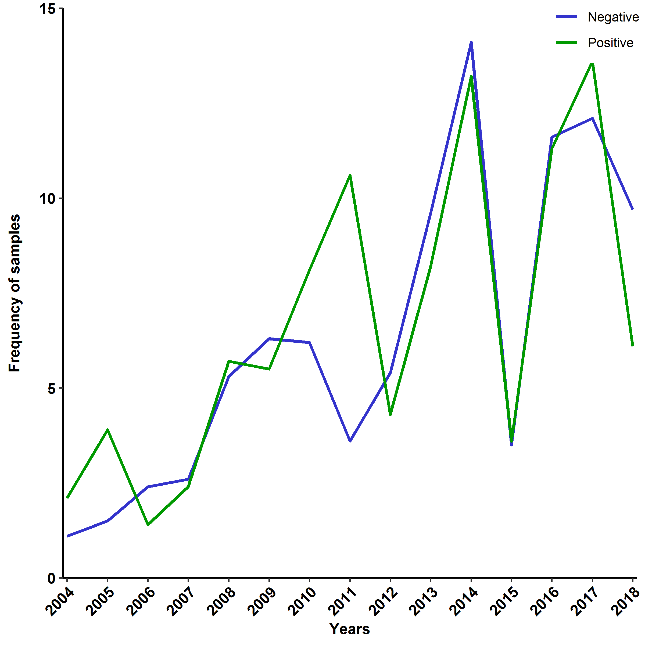


**A**

**B**


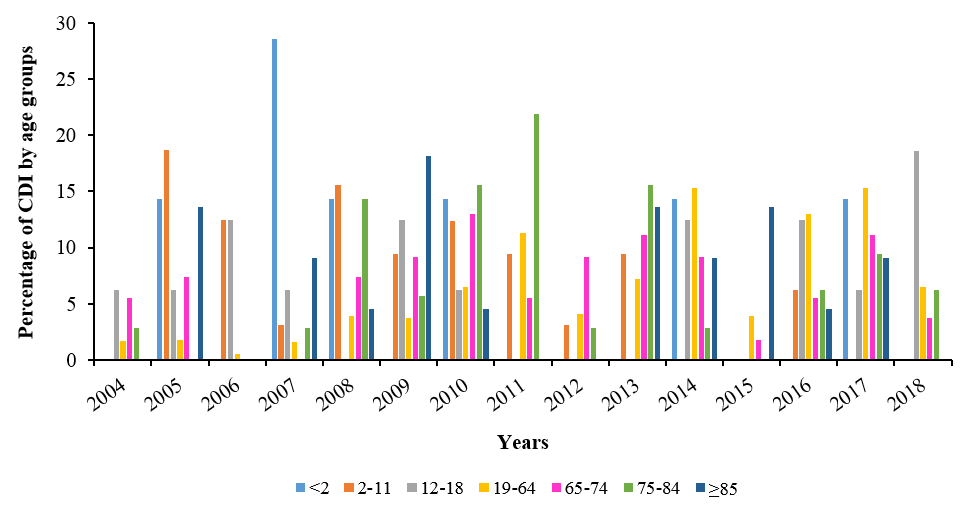


**Figure S1.** Trend of *Clostridioides difficile* infection (CDI) in Tehran over 2004 to 2018. (A) The incidence rate of CDI-positive and CDI-negative cases in Tehran over the study period (2004-2018). (B) Age-adjusted annual incidence rate of CDI during the study period.


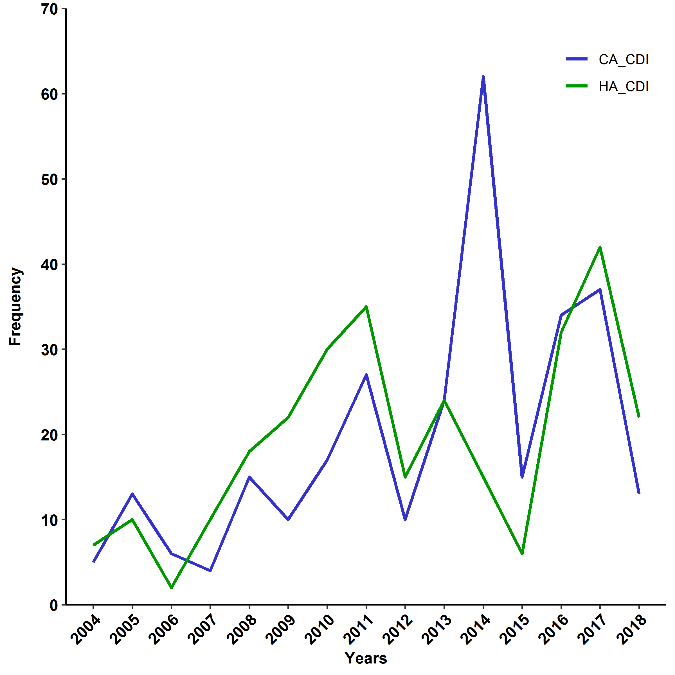


**Figure S2.** The incidence rate of HA-CDI and CA-CDI during the study period (2004-2018).


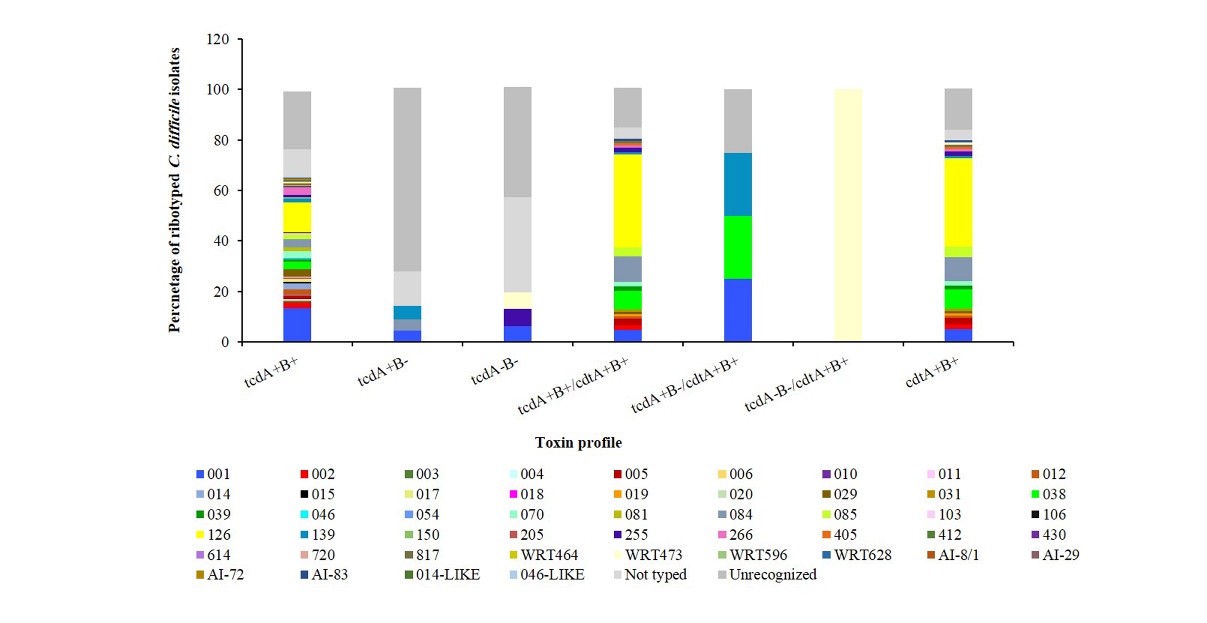


**Figure S3.** Distribution of various toxin profiles in 366 *C. difficile* ribotypes.


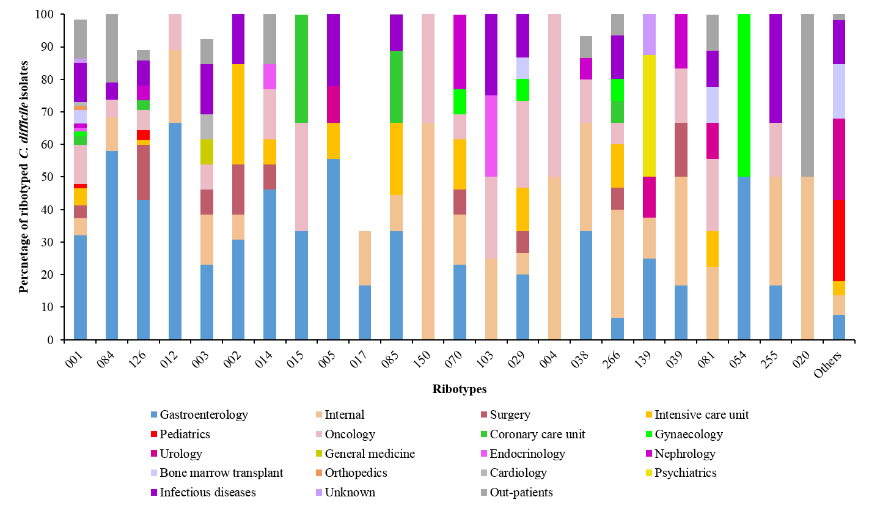


**Figure S4.** Distribution of *C. difficile* ribotypes in different hospital wards. Each hospital ward is represented by a different colour.


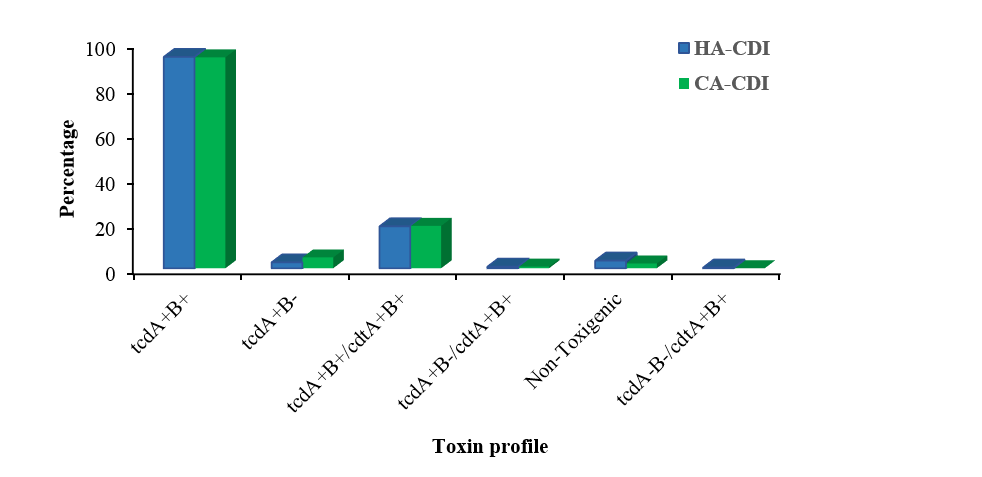


**Figure S5.** Distribution of toxin profiles of *C. difficile* isolates in HA-CDI and CA-CDI.

**
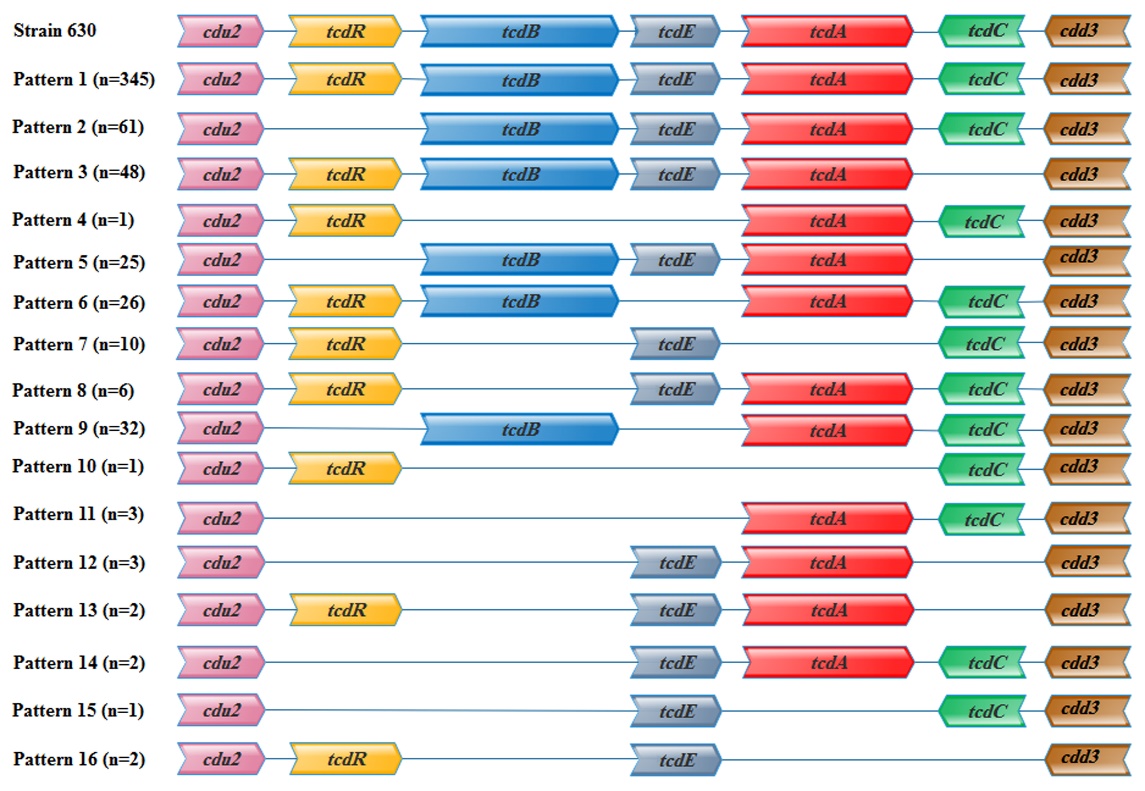
**

**Figure S6.** Genetic organization and intactness patterns of the pathogenicity locus (PaLoc) genes in 566 *C. difficile* isolates used in this study. The PaLoc genetic arrangement of *C. difficile* 630 (NC_009089.1) was used as the reference strain.
